# Cinnamic Acid Derivatives and Their Biological Efficacy

**DOI:** 10.3390/ijms21165712

**Published:** 2020-08-09

**Authors:** Ngonidzashe Ruwizhi, Blessing Atim Aderibigbe

**Affiliations:** Department of Chemistry, University of Fort Hare, Alice Campus, Alice 5700, Eastern Cape, South Africa; 201515559@ufh.ac.za

**Keywords:** cinnamic acid, antimicrobial, anticancer, antimalarial, Alzheimer’s disease

## Abstract

The role played by cinnamic acid derivatives in treating cancer, bacterial infections, diabetes and neurological disorders, among many, has been reported. Cinnamic acid is obtained from cinnamon bark. Its structure is composed of a benzene ring, an alkene double bond and an acrylic acid functional group making it possible to modify the aforementioned functionalities with a variety of compounds resulting in bioactive agents with enhanced efficacy. The nature of the substituents incorporated into cinnamic acid has been found to play a huge role in either enhancing or decreasing the biological efficacy of the synthesized cinnamic acid derivatives. Some of the derivatives have been reported to be more effective when compared to the standard drugs used to treat chronic or infectious diseases in vitro, thus making them very promising therapeutic agents. Compound **20** displayed potent anti-TB activity, compound **27** exhibited significant antibacterial activity on *S. aureus* strain of bacteria and compounds with potent antimalarial activity are **35a**, **35g**, **35i**, **36i**, and **36b**. Furthermore, compounds **43d**, **44o**, **55g–55p**, **59e**, **59g** displayed potent anticancer activity and compounds **86f–h** were active against both hAChE and hBuChE. This review will expound on the recent advances on cinnamic acid derivatives and their biological efficacy.

## 1. Introduction

**Figure 1:**
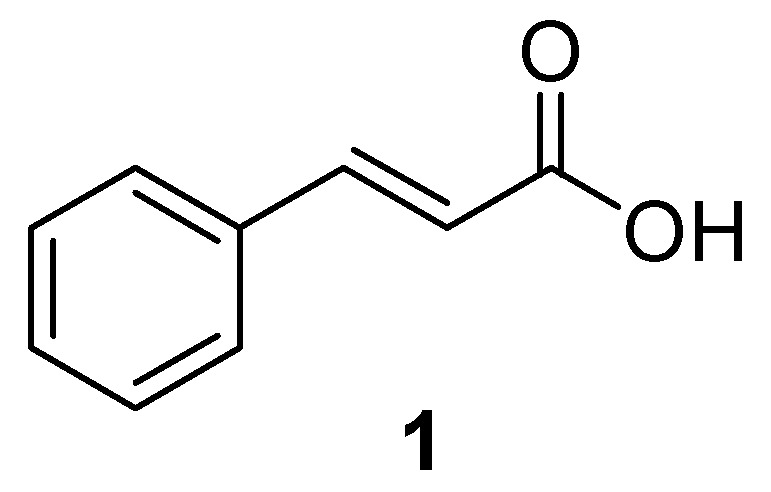
Chemical structure of cinnamic acid (**1**).

Cinnamic acid, a natural aromatic carboxylic acid [1] (Figure 1), is a key chemical found in plants such as *Cinnamomum cassia* (Chinese cinnamon) and *Panax ginseng*, fruits, whole grains, vegetables and honey [1]. The presence of an acrylic acid group substituted on the phenyl ring gives cinnamic either a cis or a trans configuration with the latter being the most common of the two [2,3]. Studies have reported that cinnamic acid exhibit antioxidant, antimicrobial [4], anticancer [5], neuroprotective, anti-inflammatory and antidiabetic properties. [6]. Cinnamic acid terminates radical chain reactions by donating electrons that react with radicals forming stable products [7]. It is also used as a fragrant ingredient in toiletries, flavorings cosmetics and detergents [2]. Cinnamic acid can be prepared by enzymatic deamination of phenylalanine [8].

Besides the cinnamic acid derivatives that occur naturally in plants [2], the presence of a benzene ring and the acrylic acid group makes it possible to modify it resulting in synthetic cinnamic acid derivatives [9]. Apart from them being intermediates in producing other compounds like stilbenes and styrenes, cinnamic acid derivatives exhibit antibacterial, antifungal, anti-inflammatory [10], neuroprotective [11], anticancer [12] and antidiabetic activities [13]. Cinnamic acid derivatives that have been reported by researchers include ferulic acid, curcumin [11], caffeic acid, p-hydroxycinnamic acid [14] coumaric and chlorogenic acids [15], etc. These cinnamic acid analogues are differentiated by the presence of hydroxyl groups on the phenyl ring that are either free or methoxylated [15].

The biological activities of different cinnamic acid derivatives have been attributed to the nature and position of the substituent groups. Drug resistance and the absence of curative therapies with low adverse side effect profiles to control cancer, microbial growth, neurological disorders etc. has led to research on the development of therapeutic compounds based on cinnamic acid [16]. Due to the biological efficacy of derivatives of cinnamic acid, this review will report different recently synthesized derivatives of cinnamic acid and their biological activity in vitro and in vivo.

## 2. Cinnamic Acid Derivatives with Antimicrobial Activity

The World Health Organization (WHO) recognizes infectious diseases caused by bacteria, viruses and fungi as a global health threat, especially in developing and poor countries. About 3.5 million people die from infectious diseases annually [17]. In 2018, the WHO reported 37.9 million people to be living with Human Immunodeficiency Virus (HIV) worldwide with 770,000 dying from AIDS-related illnesses [18]. The 2019 Global tuberculosis (TB) reported an estimated 10.0 million TB infections and 1.2 million deaths in 2018. Of the TB cases, 8.6% were people living with HIV [19]. The appearance of drug-resistant microbial strains has led to difficulty in treating diseases such as TB and malaria. Cinnamic acid derivatives are known for their antimicrobial activity and researchers are searching for antimicrobial agents which are more effective than the currently used standard drugs [20].

**Figure 2:**
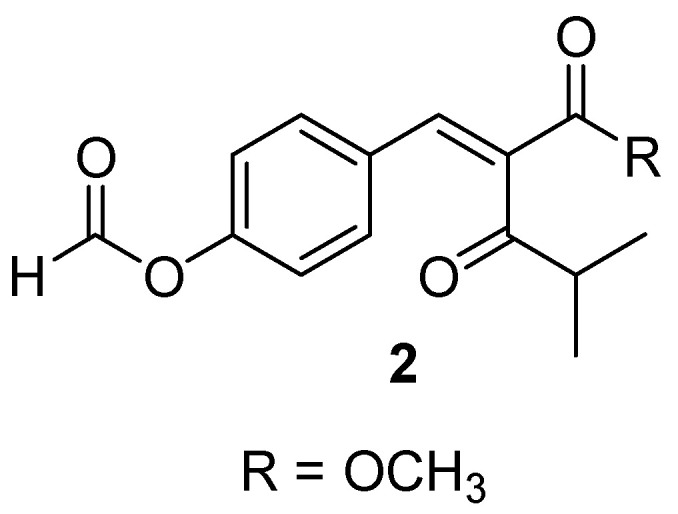
Chemical structure of 4-hydroxycinnamic acid derivative (**2**) [21].

Deng et al. extracted a 4-hydroxycinnamic acid derivative, methyl 2-{(*E*)-2-[4-(formyloxy)phenyl]ethenyl}-4-methyl-3-oxopentanoate (**2**) (Figure 2) from a plant-based endophytic fungus *Pyronema* sp. The biological activity of the compound was evaluated in vitro on a non-tuberculous mycobacterium, Mycobacterium marinum, responsible for skin infections. The compound exhibited a good inhibitory effect with an IC_50_ of 64 µM [21].

**Figure 3:**
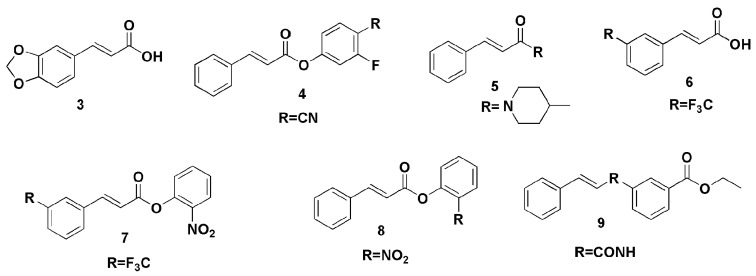
Chemical structures of cinnamic acid derivatives with antifungal activity (**3**–**9**) [22].

Korosec et al. tested antifungal activity of cinnamic acid derivatives, most of them were synthesized [22]. The antifungal activity was based on targeting CYP53A15, a unique fungal enzyme known to play a role in demethyling lanosterol, subsequently promoting fungal growth [23]. Derivatives (**3**–**9**) (Figure 3) inhibited CYP53A15 enzymatic activity against the three fungi (*Cochliobolus lunatus*, *Aspergillus niger* and *Pleurotus ostreatus*) thereby revealing the potential antifungal activity of cinnamic acid derivatives [22].

Based on the Table 1, compounds **3** and **4** were the best inhibitors of *C. lunatus* growth. The percentage fungal growth increased from compounds **3** to **9** revealing the weak antifungal activity of some of the compounds. Compound **4** was antifungal activity was potent on *A. niger* inhibitor and compound **6** was not effective in inhibiting *P. ostreatus* growth. Compound **5** exhibited broad antifungal activity in all the three fungal species. The presence of an electron-withdrawing group on the phenyl ring enhanced the antifungal activity of the compound. The type of phenyl ring substituent and its position also played an important role [23]. 2-nitro or 4-cyano substitution inhibited fungal growth to the optimum [20]. Compound **5** also exhibited the best inhibition on CYP53A15 activity making it a good candidate for the further development of antifungal drugs [22].

**Figure 4:**
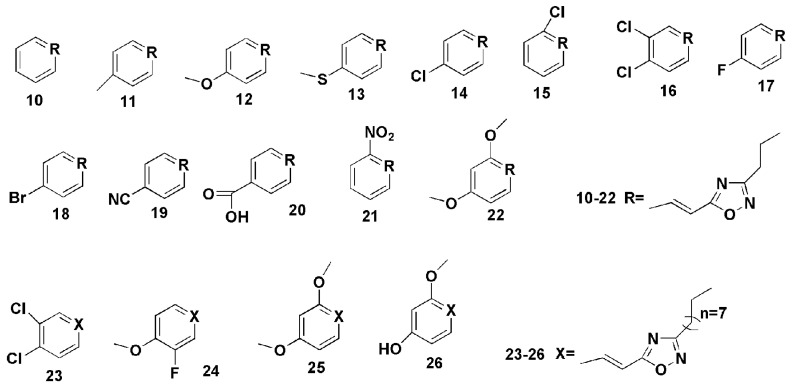
Chemical structures of the synthesized novel styryl oxadiazoles [24].

Atmaram Upare et al. synthesized cinnamic acid derivatives (**10**–**26**) (Figure 4) in which the carboxyl group was replaced with bioisostere 1,2,4-oxadiazole followed by substituting the phenyl ring of the cinnamic acid resulting in a series of novel styryl oxadiazoles. The compounds were tested in vitro at day 8 for their anti-tubercular activity against H37Ra strain of *Mycobacterium tuberculosis* (Table 2). Compound **10** was 20 times less active when compared to cinnamic acid. Compounds having electron-donating substituents on the para position of the phenyl ring (**11**–**13**) were inactive with IC_50_ > 30 µg/mL [24].

For the compounds with halogen substitutes on the phenyl ring, the position, number of substituents and the type of halogen affected the biological activity of the compounds. Para-substituted chloro-compound **14** (IC_50_ = 4.54 µg/mL) was more active when compared to ortho-substituted compound **15** (IC_50_ = 9.91 µg/mL), suggesting that the presence of chlorine on the para position enhanced the antibacterial activity of the compound [25]. Compound **17** with a 4-fluoro phenyl ring substitution was the most active of all the compounds containing fluorine with IC_50_ = 0.36 µg/mL indicating that fluoro substitution favored anti-tuberculosis activity of the synthesized compound. However, compound **24** displayed high IC_50_ value greater than 30 µg/mL suggesting the nature of the fluorine substituent was also important [24].

The introduction of an electron-withdrawing group improved their antibacterial activity (i.e., compounds **20** and **21**). Outstandingly, compound **20** with a carboxylic acid at the para position exhibited potent anti-TB activity (IC_50_ = 0.045 µg/mL) when compared to cinnamic acid. Thus, generally, the presence of an electron-withdrawing group on the para position of the phenyl ring favored significant anti-TB activity [25]. Compounds with electron-withdrawing groups exhibited improved activity (compound **23** with IC_50_ = 0.56 µg/mL was more active than compound **16** with IC_50_ = 2.35 µg/mL) while compounds with electron-donating substituents on the phenyl ring did not display a significant anti-TB activity [24].

**Figure 5:**
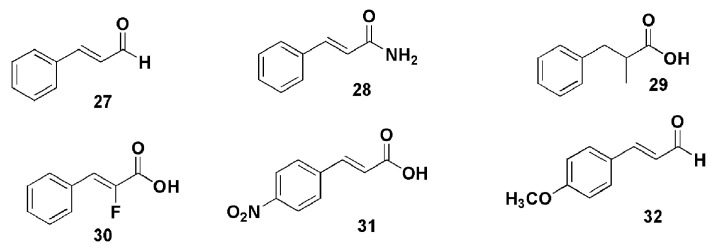
Chemical structures of compounds, **27**–**32** [16].

Malheiro et al. synthesized cinnamic acid derivatives by modifying the carboxyl group, the alkene and the phenyl ring (**27**–**32**) (Figure 5) and studied their effects, together with selected phytochemicals, on controlling planktonic *Escherichia coli*, *Staphylococcus aureus* and *Enterococcus hirae*. The phytochemicals and derivatives were also compared with the commonly used biocides such as sodium hypochlorite, triclosan, hydrogen peroxide, etc. Compound **27** appeared to be the most potent on all the three bacteria species, with better efficacy than sodium hypochlorite (Table 3) [16].

Generally, compounds **28**–**30** inhibition of bacterial growth on all the three species was not significant when compared to the control (Table 3). However, the compound **27** antibacterial effect was significant on the *S. aureus* strain of bacteria studied when compared to the control, sodium hypochlorite. The results of the compounds showed that modifications on the alkene group, phenyl ring and carboxylic acid functional groups affect the biological activity of the compounds [16].

**Figure 6:**
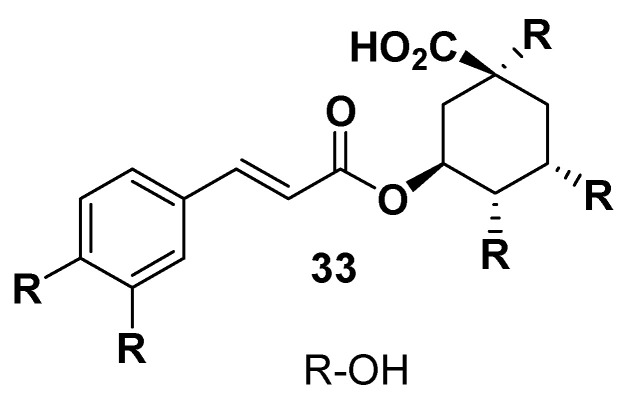
Chemical structure of chlorogenic acid (**33**) [26].

Antimicrobial activities of chlorogenic acid (compound **33**) (Figure 6) include antibacterial activity against multi-drug resistant bacteria, antifungal and anti-viral activities against HSV-1 and HSV-2, adenovirus, Ebola and HIV. Antifungal effects of compound **33** are known as it is effective against *Candida albicans* through impacting the cell membrane. It is effective against *Escherichia coli*, *Staphylococus aureus*, *Staphylococcus epidermidis* and *Klebsiella pneumonia*, among other bacteria. It has broad applications in food due to its insensitivity against probiotic bacteria [26].

**Figure 7:**
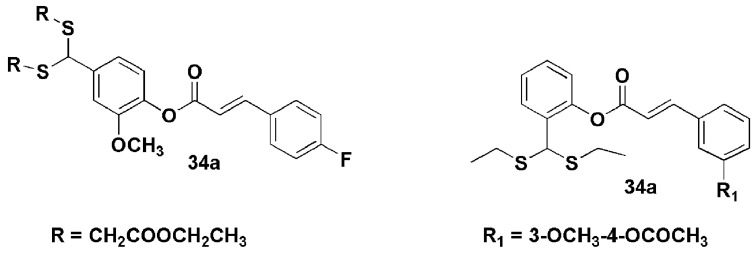
Cinnamic acid derivatives with dithioacetal moieties (**34a** and **34b**) [27].

Wang et al. evaluated anti-Tobacco mosaic virus (TMV) activities of synthesized cinnamic acid derivatives containing dithioacetal moieties. Only **34a** and **34b** (Figure 7) displayed curative activities of 82.6% and 83.5%, respectively which were significant when compared to ribavirin (70.3%) and xiangcaoliusuobingmi (71.7%), used as the control [27].

Compounds **34a** and **34b** antiviral activities were comparable to the activity of ningnanmycin (85.2%) used as the control. Treatment of TMV particles with **34b** caused serious damage and fracture. Molecular docking results showed that **34b** bonded excellently with amino acid residues GLN34, THR37, ARG90, and ARG46 of TMV coat protein via hydrogen bonds [27].

Despite the several reports on cinnamic acid derivatives, the compounds with potent antimicrobial activities are **3**–**5** which were effective against fungal strains such as *C. lunatus*, *A. niger* and *P. ostreatus* when compared to cinnamic acid [22]. Furthermore, compounds with high antibacterial activity are **20** which exhibited potent anti-TB activity when compared to cinnamic acid [24] and compound **27** which was effective on *E. coli*, *S. aureus* and *E. hirae* when compared to sodium hypochlorite [16].

## 3. Cinnamic Acid Derivatives with Anticancer Activity

In 2018, WHO reported 18.1 million new cancer cases and 9.6 million deaths making cancer one of the leading causes of death [28]. The high number of people who die from cancer has led to researchers making unremitted efforts to develop safe and efficient antitumor agents [29]. The effectiveness of most of the currently used chemotherapeutic agents is severely limited by drug resistance [30]. Most drugs fail during invasion and metastasis of cancers making patients succumb to the disease [30]. Cinnamic acid derivatives have anticancer effects and are effective against a wide range of cancers such as breast [5] colon, lung, etc. Antiproliferative activity of cinnamic derivatives against tumors has been reported [31]. Cinnamic acid derivatives, such as cinnamaldehyde, use apoptosis, among other ways, to destroy cancerous cells [32].

**Figure 8:**
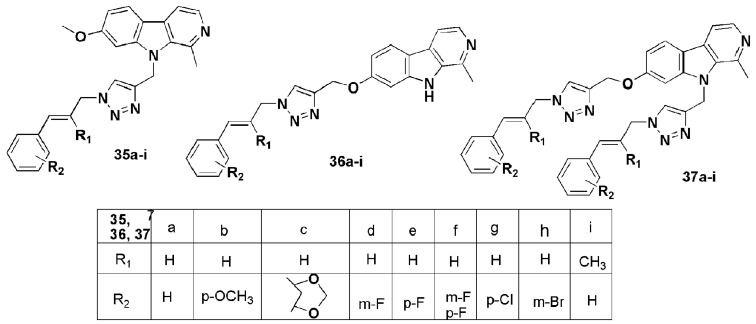
Structures of cinnamic acid-harmine hybrids (**35**–**37**) [33].

Perkoic et al. synthesized harmicine and cinnamic acid hybrids (**35**–**37**) (Figure 8). In vitro cytotoxicity of the hybrids on a human liver hepatocellular carcinoma cell line (HepG2) depended on the harmicine type and substituent in the cinnamic acid derivative. Compounds **36d**, **36e** and **36f** were the most effective compounds against HepG2 cell line with IC_50_ values of 3.11, 2.19 and 0.74 µM, respectively, when compared to harmine with IC_50_ greater than 250 µM [33]. The increased cytotoxicity of compounds **36d**, **36e** and **36f** is attributed to them being *O*-harmicines while the others are *N*-harmicines and *N,O*-bis-harmicines. However, no specific structure–activity relationship was reported [33].

Pellerito et al. synthesized ferulate derivative, tributyltin(IV) ferulate. In vitro studies on colon cancer cells, HCT116, Caco-2 and HT-29 showed that the ferulic acid did not alter the viability of any of the three cell lines even up to 1 µM concentration while tributyltin(IV) ferulate showed remarkable cell viability reduction at 400 nM (−78.9% for HCT116, −64.1% for Caco-2 and −57.5% for HT-29). Tributyltin(IV) ferulate induced autophagic cell death and arrested the cell cycle thereby reducing colon cancer cell viability. Thus, apart from it showing remarkable cell viability against the tested cell lines, it also showed a very significant effect at very low doses making the novel ferulic acid derivative a promising colon cancer therapy candidate [34].

**Figure 9:**
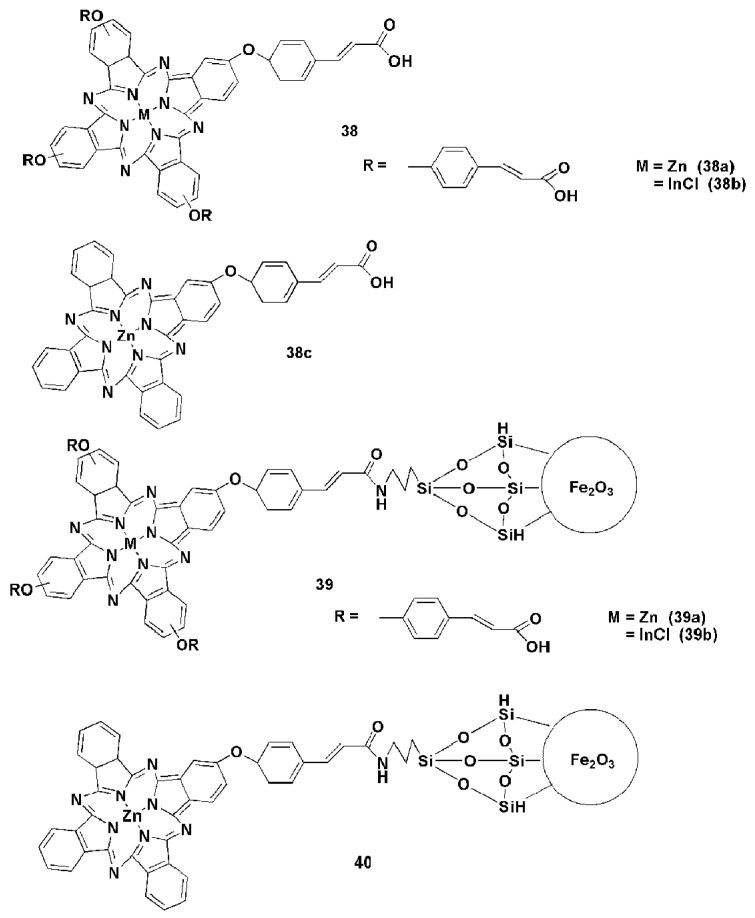
Chemical structures of zinc and indium tetra cinnamic acid phthalocyanine (Pc) complexes covalently linked to amino-functionalized magnetic nanoparticles (AMNPs) [35].

Matlou et al. synthesized zinc and indium tetra cinnamic acid phthalocyanine (Pc) complexes (**38**–**40**) (Figure 9) which were covalently linked through an amide bond to amino-functionalized magnetic nanoparticles (AMNPs) [35]. Cinnamic acid was connected to the nanoparticles due to its antitumor activity [36]. Compounds **38a–c** were compared with compounds **39a–c** for photodynamic therapy and in vitro cytotoxicity against human breast adenocarcinoma cell lines. Compounds **38a**, **39a**, **38b** and **39b** triplet quantum yield improved after linking to AMNPs but decreased for **39c** (0.65) and **38c** (0.73) due to photoisomerization on the alkene double bond of cinnamic acid [37]. The triplet lifetimes for complex **40** was shortened from 258 µs to 224 µs due to triplet energy lowering and non-radiative decays increasing after linking AMNPs to the complex. Singlet oxygen quantum yields also increased in the presence of AMNPs for **38a** and **39a** and decreased for **40**.

All the six compounds showed very low in vitro cytocidal effects in the dark with compound **38c** showing the least cell viability at 78% at 80 µg/mL. This indicated that the used drug have low dark cytotoxicity. Photodynamic activity (PDT) of compounds **38a–c** on MCF-7 cells increased as the drug concentration increased from 5–80 µg/mL and the cell death was evaluated based on the cell confluence of viable cells. PDT activity decreased for compounds **39a–c** since phthalocyanine photoactivity decreased upon linking to AMNPs due to phthalocyanine complex aggregation. Compound **38b** outperformed **38a** and **38c** due to spin-orbit coupling and efficient transfer of energy to ground state molecular oxygen, which gives it the significant cytocidal activity against the tumorigenic cells [35].

**Figure 10:**
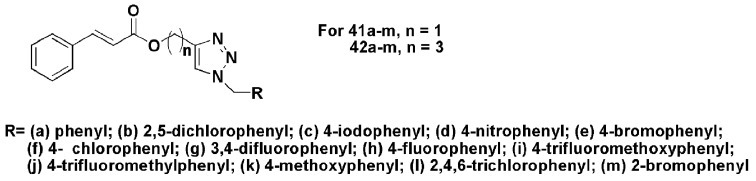
Chemical structures of compounds **41a–m** and **42a–m** [38].

Lima et al. synthesized cinnamic acid derivatives having 1,2,3-triazolic moiety (**41a–m** and **42a–m**) (Figure 10) and evaluated their antimetastatic activity. At 100 µmol/L, The compounds **41a**, **41b**, **41f**, **41h–j**, **41l**, **41m**, **42a–e**, **42g–I** and **42k–m** significantly suppressed B16-F10 cell migration when compared to the cells treated with 0.4% DMSO. Compound **42a** was the most effective with cell migration suppression of 86% when compared to the control. B16-F10 cell proliferation was also significantly impaired by compound **42a**. There was a decrease in B16-F10 cell invasion and adhesion upon treatment of compound **42a** for 24 h which confirmed the antimetastatic activity of cinnamic acid derivatives against colon carcinoma and human lung adenocarcinoma cells [38].

B16-F10 cell colonies were reduced when treated with different concentrations of compound **42a** at 12.5 μmol/L (25.5%), 25 μmol/L (20.6%) and 50 μmol/L (93.1%) when compared to the control. Since cell proliferation, migration, invasion, adhesion and colonization are the central steps in the in vivo metastasis process [39], the positive results exhibited by compound **42a** suggest its efficacy in reducing B16-F10 cells metastatic potential. Vero and fibroplast (NIH3T3) cells appeared somewhat sensitive to compound **42a** at 100 µmol/L and no hemolysis was significant at different concentrations. Molecular binding prediction indicated that the compound **42a** inhibitory ability is due to its interaction with MMP-9 and MMP-2, enzymes involved directly in melanoma progression [40] The heteroatoms in compound **42a** have the capability to form hydrogen bonds with proteins thereby inhibiting the two enzymes [10].

**Figure 11:**
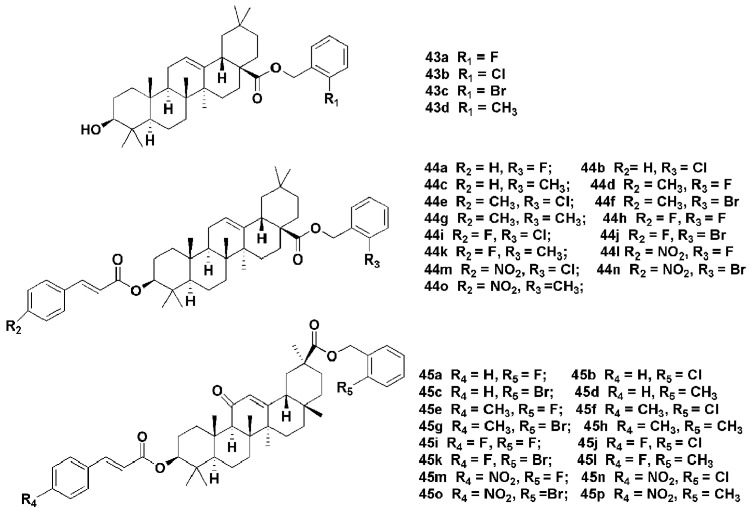
Chemical structures of the novel novel oleanolic acid (OA)-cinnamic acid ester derivatives (**43a–d** and **44a–o**) and glycyrrhetinic acid (GA)-cinnamic acid ester derivatives (**45a–p**) [5].

Wang et al. designed and synthesized novel oleanolic acid (OA)-cinnamic acid ester derivatives (**43a–d** and **44a–o**) and glycyrrhetinic acid (GA)-cinnamic acid ester derivatives (**45a–p**) (Figure 11) and assessed their in vitro cytotoxicity against MCF-7 (breast cancer), HeLa (cervical cancer) and L-O2 (a normal hepatic cell) [5].

Based on the Table 4 below with selected compounds, compound **45c** exhibited the most significant cytotoxicity on L-O2 cells. The strong inhibitory activity (1.79 µM) against MCF-7 cells shown by compound **44e** suggests that linking 4-methylcinnamic acid to the C-OH position of OA-2-chlorobenzyl ester has the ability to significantly improve the inhibition of MCF-7 cells. Compound **44o** was the most potent compound against HeLa cells with IC_50_ of 1.35 µM, close to that of the control, gefitinib (1.28 µM) [5].

Compounds **44a**, **44h** and **44f** could not inhibit any of the three cell lines. The structure–activity relationships showed no clear trend when the substituent on the benzyl of GA was either electron-donating or electron-withdrawing. However, the electron-donating group (CH_3_) on the ortho position of the benzyl group on OA increased the inhibitory effect of the compounds on HeLa cells. Compounds **43d** and **44o** were further evaluated due to their cytotoxic effect against HeLa cells and they were found to induce apoptosis and increased reactive oxygen species levels in a dose-dependent manner. Both compound **43d** and **44o** induced autophagy in HeLa cells [5].

**Figure 12:**
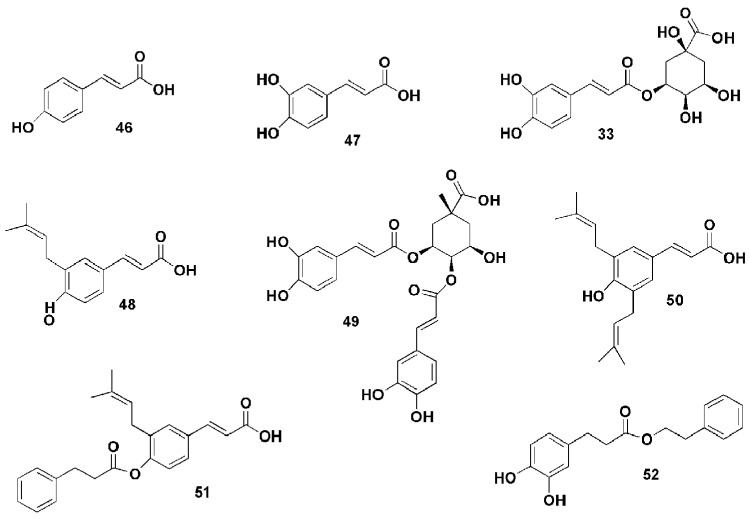
Chemical structures of the cinnamic acid derivatives in propolis components [41].

Endo et al. examined autophagy-inducing activity of cinnamic acid derivatives in Brazilian green propolis: p-coumaric acid **(46)**, caffeic acid **(47)**, chlorogenic acid **(33)**, drupanin **(48)**, 3,4-caffeoylquinic acid (3,4-CQA) **(49)**, artepillin C (ArtC) **(50)**, baccharin **(51)** and caffeic acid phenethyl ester (CAPE) **(52)** (Figure 12) by subjecting them to castration-resistant prostate cancer (CRPC) CWR22R1 cells. Compounds **33**, **46**, **47**, and **48** were less cytotoxic but compounds **49**–**52** significantly decreased prostate cancer [41].

CWR22Rv1 cells viability after 24 h treatment revealed compound **52** to be the most potent, even killing 90% of the cells at 30 µM dosage. Compounds **50** and **51** significantly increased LC3-II levels, showing high autophagy-inducing activity and compound **50** was more potent than **51**. Further examinations showed that compound **46** induced autophagy through mechanisms different from lysosomal enzymes inhibition [41]. Choudhari et al. also reported the in vitro potency of Indian stingless bee propolis as anticancer agents [42]. Treatment of A549 cells with compound **52** (50 µM) significantly decreased, in a dose-dependent manner, the expression of claudin-2, a protein highly expressed in most cancer cells [43]. Compound **52** up-regulated lκB levels and inhibited lκB phosphorylation with no effect on Akt, thus decreasing the transcriptional activity of claudin-2 in A549 cells. Compound **52** enhanced the sensitivity of the cells to anticancer agents such as doxorubicin by increasing the paracellular permeability to anticancer agents. Shimizu and Suzuki reported the ability of compound **52** to reduce the inflammation stage in macrophages, supporting the results of Endo and his colleagues [44]. Positive results have been reported for bioavailability and pharmacokinetics of compound **52** in rodents but they still remain unclear in humans [45].

Cotreatment of CWR22Rv1 cells with compound **50** and wortmannin or U0126, which are autophagy inhibitors, greatly decreased the viable cells and significantly increased lactose dehydrogenase release when compared to treating with compound **50** alone indicating the enhancement of the cytotoxic potency of compound **50** by autophagy inhibition. The authors also reported the involvement of necroptosis in the cell death when treated with compound **50** and autophagy inhibitor. Thus based on their findings, compound **50** induces autophagy and apoptosis in CWR22Rv1 cells and most importantly, autophagy inhibition further increases apoptosis and induces necrosis [41].

**Figure 13:**
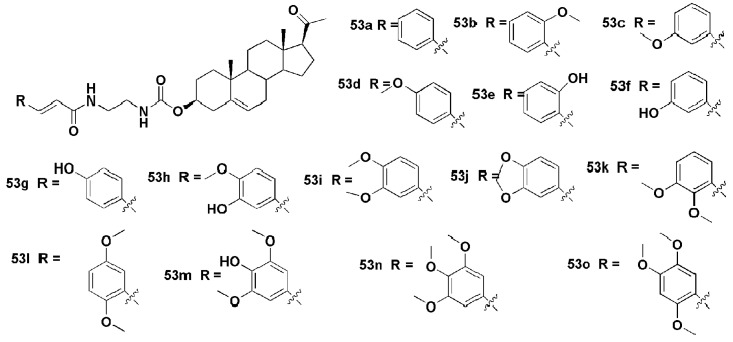
Chemical structures of designed and synthesized novel cinnamic acid-progenone hybrids (**53a–o**) [31].

Ge et al. designed and synthesized novel cinnamic acid-progenone hybrids (**53a–o**) (Figure 13) and evaluated their biological activity as potential antiproliferative agents against A549, H157, HepG2, MCF-7, and HL-60 cell lines (Table 5) [31].

Compounds **53f** and **53g** were the most potent compounds against all the five cancer cell lines (Table 5). Based on the IC_50_ values of compounds **53b–53g**, 3-OH or 4-OH increased the potency of the compounds while methylation of the hydroxyl or changing its position decreased the compound activity especially towards A549 and HL-60 cancer cell lines. Antiproliferative activity also decreased upon introducing another methoxyl group (**53h**) or dialkylation of hydroxyl **(53i–53l)** indicating the role played by 3-OH and 4-OH on the phenyl ring on the compound’s activity which was observed in **53m** being more active than **53n** and **53o** [31].

At 50 µM, all the compounds exhibited inhibition less than 50% on two non-tumor cell lines, BEAS2B and MCF-10A, thus the hybrids were considered to be selectively toxic towards the cancer cell lines compared to the non-tumor cells. Cell cycle results showed **53f** arrested the cell progression in G1 phase and enhanced apoptosis in A549 cells, the regulation of Bax/Bcl-2 expression by **53f** contributing to the latter [31].

**Scheme 1:**
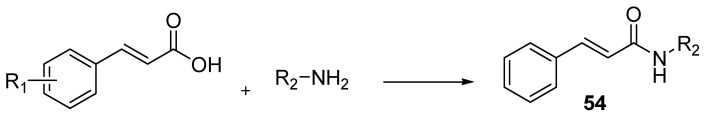
General synthetic route for cinnamamide. Reagents and conditions: POCl_3_, TEA, DCM, 0 °C [25].

After reviewing several cinnamamides and their structure–activity relationships in anticancer activity, Gaikwad et al. deduced that the compounds were more active when the ortho group remained unsubstituted but electron-donating groups were tolerated (Scheme 1). Electron-withdrawing groups such as NO_2_, CN and CF_3_ at the para position were important for potency and selectivity with *tert*-Bu and OCH_3_ being exceptions since they are electron-donating groups contributed to the activity of the compounds. The presence of electron-donating groups such as OCH_3_, vinyl and NH-R at the meta position was preferred for anticancer activity. For the R_2_ substitution, heterocyclic and aromatic amines significantly elevated anticancer activity while aliphatic amines showed relatively low potency [25].

**Figure 14:**
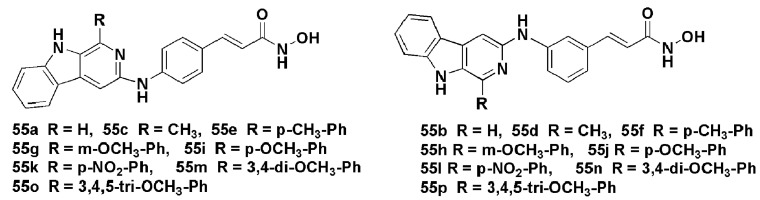
Chemical structures of compounds **55a–p** [30].

Most anticancer agents used to treat hepatocellular carcinoma suffer from drug resistance [46]. In order to overcome drug resistance, Ling et al. developed β-carboline and *N*-hydroxycinnamamide compounds (**55a–p**) (Figure 14) [30]. All the compounds, **55a–p**, inhibited histone deacetylase 1 (HDAC1), a key HDAC subtype shown in cancer cell differentiation and proliferation [30]. Compound **55p** displayed the best potency with IC_50_ of 1.3 Nm when compared to suberoylanilide hydroxamic acid (SAHA) (IC_50_ = 142 nM), an approved HDAC inhibitor. The presence of an electron-donating group such as the R group such as methoxyl (**55g–j**) or more than one methoxyl groups (**5m–p**) enhanced inhibitory potency compared to compounds with a hydrogen (**55a,b**) or methyl (**55c,d**) [30].

Antiproliferative activities of the compounds against human colon cancer cells (HCT116), human hepatocellular carcinoma (HCC) cells (HepG2 and SMMC-7721), and human lung cancer cells (H1299) showed that **55e**, **55g–I** and **55o,p** (IC_50_ = 0.41–4.29 µM) showed better antiproliferative potency when compared to SAHA (IC_50_ = 4.79–7.02 µM) (Table 6). Compound **55p** displayed the best antiproliferative effect against drug-sensitive Bel7402 and drug-resistant Bel7402/5-FU with IC_50_ values of 0.85 and 2.09 µM, respectively. Compound **55p** induced better Bel7402/5-FU cell apoptosis than SAHA together with autophagic flux activity in Bel7402/5-FU cells by down-regulating p62 and LC3-1 and up-regulating LC3-ΙΙ proteins, making it a potential candidate in developing anticancer agents to treat multi-drug resistant human cancer [30].

**Scheme 2:**
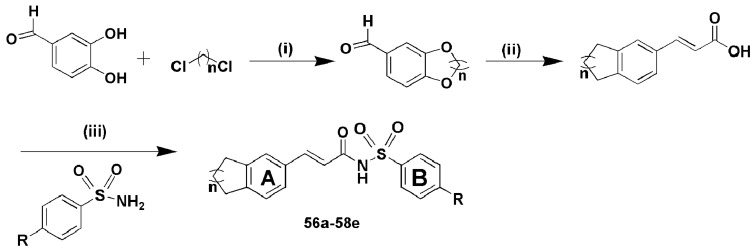
General synthesis of cinnamic acid sulfonamide derivatives (**56a**–**58e**). Reagents and conditions: (i) K_2_CO_3_, acetone, reflux, 29 h; (ii) piperidine, pyridine, 80–90 °C, 24 h; (iii) substituted benzene-sulfonamides, Dichloromethane (DCM), 1-Ethyl-3-(3-dimethylaminopropyl)carbodiimide (EDCI), 4-Dimethylaminopyridine (DMAP), overnight [47].

Substituted cinnamic acyl sulfonamide derivatives (**56a–58e**) (Scheme 2) were synthesized and screened for their antiproliferative activity on MCF-7 human breast cancer cells. Based on Table 7, compound **56a** displayed the best activity (IC_50_ = 0.17 µg/mL) when compared to the control, colchicine [47].

Compound **56a** had no substituent on B phenyl ring but had a benzdioxan group on the A ring meaning the presence of a substituent on the B phenyl ring decreased the antiproliferative activity of the compound. Tubulin polymerization assay showed that compound **56a** was the most potent compound at inhibiting tubulin polymerization in vitro. Molecular docking revealed that compound **56a** formed one hydrogen bond and Van der Waals forces with tubulin [47].

**Figure 15:**
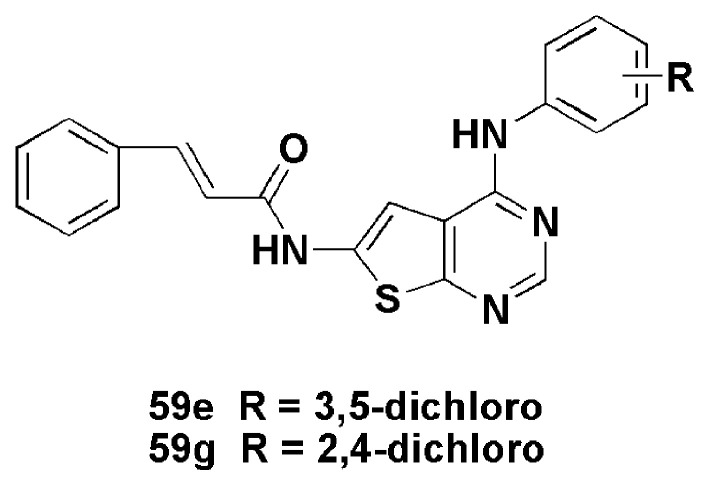
Structure of compounds **59e** and **59g** [48].

Toolabi et al. synthesized 6-Cinnamoyl-4-arylaminothienopyrimidines and compounds **59e** and **59g** (Figure 15) were very effective against A549 and HeLa cell lines [48]. Compound **59e** exhibited in vitro antiproliferative activity against both A549 and HeLa cell lines with IC_50_ values of 0.04 and 0.004 µM, respectively. Compound **59g** had an IC_50_ of 0.033 µM against HeLa cells. Both compounds were better than the reference drug, erlotinib with IC_50_ values of 3.80 and 7.48 µM against A549 and HeLa cells, respectively. Compound **59e** was further found to inhibit EGFR and ERH1/2 phosphorylation in HeLa cell lines [48].

**Figure 16:**
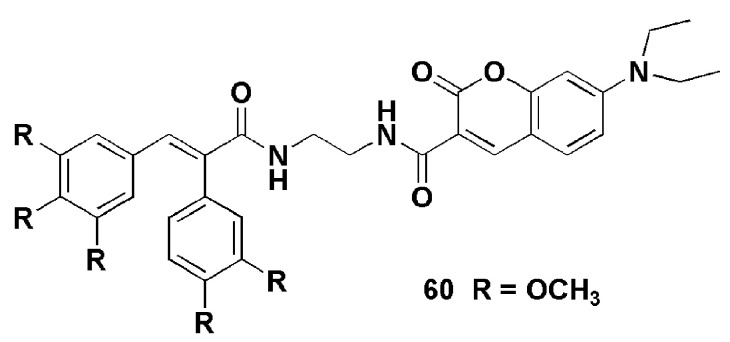
Chemical structure of compound **60** [49].

Yang et al. synthesized a number of cinnamic acid derivatives and found only compound **60** (Figure 16) which exhibited 25.78% cell viability when tested against leukemia (HL-60) cells at 20 µM for 48h [49]. The structure–cytotoxic activity relationship revealed that the presence of a trimethoxyl group on the phenyl ring, an ethylenediamine linker, inserting aryl substitutes at the C-α position and the presence of α,β-unsaturated ketone carbonyl substituent are all beneficial for the cytotoxicity of the compound. Compound **60** showed better uptake in HL-60 cells than in normal cells, with the mitochondria being the main site of accumulation. At 10 and 15 µM, compound **60** mainly induced apoptosis while at 20 µM it was elicited necrosis of the HL-60 cells [49].

The potent derivatives with anticancer activities which need further studies are **36d**, **36e**, **36f**, **43e**, **56a**, **43d**, **44o**, **55e** and **55g–p**. Cinnamic acid derivatives with potent anticancer activity against liver cancer are compounds **36d**, **36e** and **36f** [33]. Compound **43e** and **56a** were potent on MCF-7 cell lines in vitro [5,47] and compounds **43d**, **44o**, **55e** and **55g** were active against cervical cancer cell lines when compared to other derivatives which have been reported [5,48]. Compounds **55g–55p** were effective on human hepatocellular carcinoma cell line drug resistant cancer cell lines [30].

## 4. Cinnamic Acid Derivatives with Antiparasitic Activity

Parasitic diseases such as *Psoroptic* acariasis, malaria and leishmaniasis remain a major threat to public health especially to millions of people living in poor tropical countries [50]. In 2018, an estimated 405,000 people died of malaria, a parasitic infection calling for more measures to reduce the number of fatalities [51]. Resistance to broad spectrum anthelmintics in livestock helminths is a global threat to livestock such as sheep and goats [50]. The effects caused by these parasitic diseases on humans and livestock has led to the development of cinnamic acid derivatives with antiparasitic activity.

Perkovic et al. synthesized hybrids of harmine and cinnamic acid (**35a–37i**) (Figure 8) for antiplasmodial evaluation against the erythrocytic stage of *Plasmodium falciparum* (chloroquine-sensitive Pf3D7 and chloroquine-resistant PfDd2 strains) and hepatic stage of *P. berghei* as well as cytotoxicity against HepG2 cell line. From Table 8, compounds **36c**, **36g**, **36h** and **37b** (IC_50_ = 0.13–0.16 µM) were the most active against Pf3D7 strain with **37b** exhibiting the most potency against *Pf3D7* and *PfDd2*. Results on harmicines activity against *P. berghei* showed that they were more effective with (IC_50_ 0.4–2.62 µM) when compared to the reference drug, primaquine (IC_50_ = 8.4 µM). Conjugating harmine with cinnamic acid derivatives via triazole linker produced hybrids with excellent antiplasmodial activity when compared to the parent compounds at the erythrocytic and hepatic stages of *Plasmodium* infection [33].

Structure–activity relationship showed that the general activity pattern was **36** ˃ **37** ˃ **35** except for the p-OCH_3_ substituted series which had **37b** as the most potent compound. For series **35** and **37**, the bulkiness of R_2_ enhanced their antiplasmodial activity in vitro. Replacing hydrogen with fluorine on the m- and p- positions of the cinnamic acid ring resulted in compounds with enhanced antiplasmodial activity for series 35 and 36 (for example **35e** ˃ **35a**, **35d** ˃ **35a**) but not for series 37 [33].

**Figure 17:**
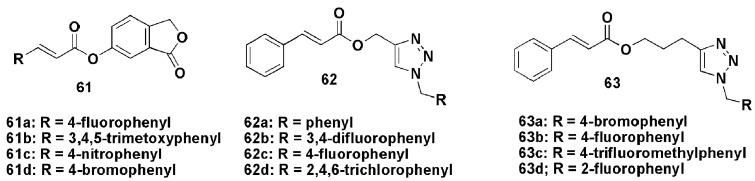
Chemical structures of compounds **61**–**63** (**a–d**) [3].

Rodrigues et al. synthesized cinnamic acid derivatives (**61**–**63**) (Figure 17) and tested their leishmanicidal activity against promastigote and amastigote forms of *Leishmania braziensis*. At 10 µM, all the derivatives with an isobenzofuranone moiety (compounds **61a–d**) were toxic to promastigotes with survival less than 75%. Compounds **62a–d** were not toxic to promastigotes of *L. braziliensis*. Compounds with a triazolic moiety (**63a–d**) revealed that only compound **63a** and **63c** were toxic with 80% and 75% survival. Compound **63b** was the most active and its activity was similar to the positive control, amphotericin B (3.125 µg/mL) (Table 9) [3].

When treated against amastigote forms, compounds **61b**, **62b** and **62d** were the most active with 80.47%, 86.32% and 80.12% toxicity, respectively, which was lower than amphotericin B (98.25%). Compounds **61b** and **62b** were further assayed because of their non-toxic nature to macrophages and their IC_50_ values were 7.58 µM and 3.05 µM, respectively, when tested in vitro on the promastigote forms [3].

These findings suggest that the presence of an electron-withdrawing group on the phenyl ring of cinnamic acid enhanced the compound’s anti-leishmanicidal activity. Halogenation of the phenyl ring of the triazole moiety plays an important role on the anti-leishmanicidal activity of the compounds. The results showed that the combination of two different bioactive chemical groups can yield effective novel anti-leishmanicidal drugs [3].

**Figure 18:**
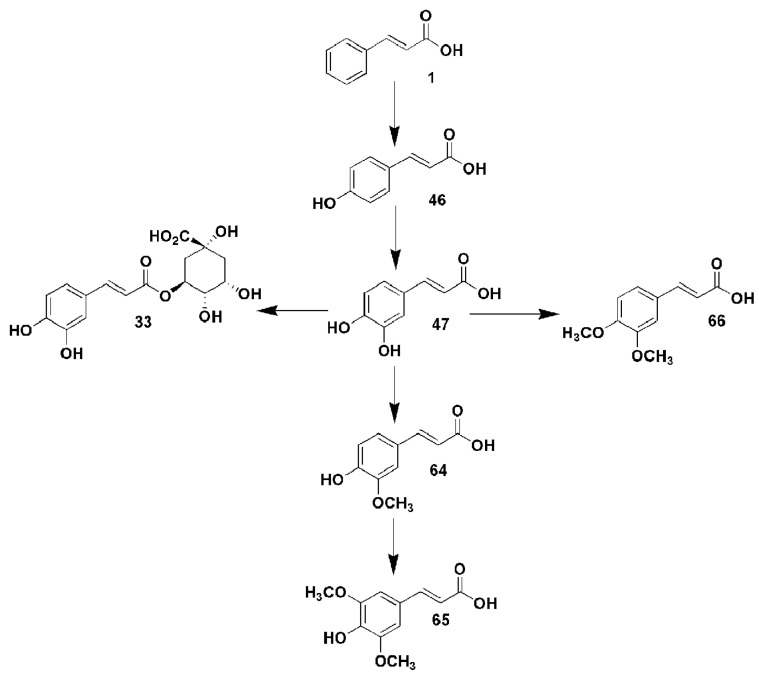
Chemical structures of cinnamic acid with in vitro anthelmintic (AH) activity [15].

Mancilla-Montelongo et al. evaluated the in vitro anthelmintic (AH) activity of cinnamic acid (**1**) and its six derivatives (p-coumaric acid (**46**), caffeic acid (**47**), trans-ferulic acid (**64**), trans-sinapic acid (**65**), 3,4-dimethoxycinnamic acid (**66**) and chlorogenic acid (**51**) (Figure 18) against the larvae and eggs of a gastrointestinal nematode, *Haemonchus contortus*. Only ferulic acid and chlorogenic acid showed notable AH activity ˃74% and ˃39% egg inhibition, respectively, at 300 µg/mL. Although compounds **1**, **46** and **47** showed no egg hatching inhibition even at 400 µg/mL, they damaged the larvae structurally, leading to their mortality [15].

The authors also made mixtures (M) of the analogues. M1 was a combination of all the seven analogues. M2 was a combination of compounds **64** and **33** which blocked larval hatching. M3 was a combination of compounds **1**, **46**, **64**, **65** and **33**. M4 was a combination of compounds **1**, **46** and **65** and the combination resulted in the larval damage. M5, which is a combination of compounds **1** and **46**, exhibited significant ovicidal activity. M1 exhibited an effective concentration 50% (EC_50_) of 1628.10 µg/mL, M4 (1143.75 µg/mL) and M3 (923.37 µg/mL). M2 and M5 showed no significant EC_50_ value revealing an antagonistic effect. The presence of two free hydroxyl groups in compound **47** did not produce any significant effect of the compound active against *H. contortus* eggs suggesting that the activity of the compounds is based on their ability to inhibit hatching-related processes and their ability to form a complex film by binding to the proteins [15].

**Figure 19:**
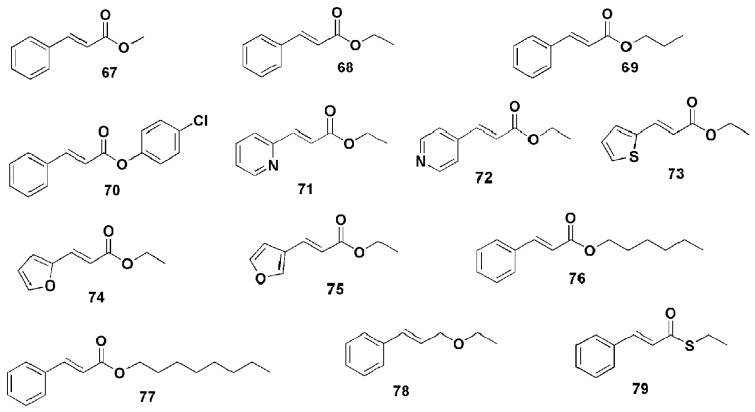
Chemical structures of compounds **67**–**79** [52].

Chen et al. synthesized a series of cinnamic acid derivatives (**67**–**79**) and evaluated their efficacy against *Psoroptes cuniculi*, a non-burrowing ear mite causing *Psoroptic* acariasis (Figure 19). The synthesized cinnamic acid derivatives included thiol esters, esters, ketones, amides and analogues with a heteroaromatic ring. The structure–activity relationship of the derivatives was studied. Compounds exhibiting higher activity were further studied for median lethal concentrations (LC_50_) and median lethal times (LT_50_) in comparison with the reference drug, ivermectin [52].

Based on Table 10, compounds **67**–**75** and **79** had a mean mortality of 84% and above with only compounds **77** and **78** exhibiting mean mortality below 40% at 0.5 mg/mL. These compounds were potent when compared to ivermectin, an acaricidal agent due to factors such as lower molecular weight and simplified structure, affordability and non-toxicity to mammals and higher activity [52,53]. All the compounds displayed LC_50_ values higher than ivermectin.

The cinnamic acid esters (**67**–**70**), the type of alkyl group, the length of the chain and the degree of branching greatly affected their biological activity. The compounds with potent biological activity were compound **68**, **71**, **73** and **74**. Steric hindrance close to the ester group decreased the activity of the compounds. The conversion of a carbonyl on the ester group to a methylene group led to a notable decrease in activity as seen when comparing **68** with **78**. Activity decreased when ester (**68**) was changed to thiol ester (**79**). Replacing the phenyl group (**68**) with 2-pyridinyl (**71**) or 2-furanyl (**74**) increased the activity [52]. Based on the different reports on the antiparasitic activity of cinnamic derivatives, the potent cinnamic acid derivatives with antimalarial activity are **36c**, **36g**, **36h** and **37b** [33].

## 5. Cinnamic Acid Derivatives with Neurological Activity

The brain, an organ of soft nervous tissue is contained in the skull of vertebrates. It functions as the coordinating centre of sensation, intellectual and nervous activity [54]. When the brain is damaged, it can affect many different things, including memory, sensation, and even personality. A number of brain diseases and neurological disorders have been found which include brain tumor, traumatic brain injury and Alzheimer’s disease, and these need to be treated for the well-functioning of the brain [55]. The treatment of Alzheimer’s disease is of prime importance since the disease is characterized by memory loss, cognitive impairment and can be fatal. Treatment methods include restoring acetylcholine levels [56] and clearing the neurotoxic amyloid-beta protein [1]. Research has proven that cinnamic acid derivatives have neuroprotective ability and scientists keep on looking for neuroprotective agents based on cinnamic acid with better biological activity [6].

**Scheme 3:**
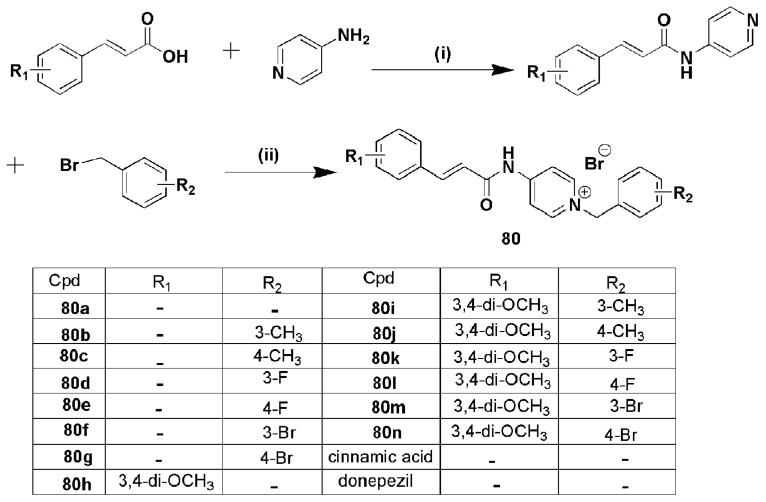
Synthetic route for compounds 80a–n. Reagents and conditions: (i) DMAP/EDCl, DCM, rt, 12 h; (ii) CH_3_CN, reflux, 1–3 h [11].

Lan et al. reacted *N*-benzyl pyridinium with cinnamic acids of different substitutions to obtain cinnamic acid derivatives (**80a–n**) (Scheme 3) that inhibit cholinesterase, suggesting potential efficacy in treating Alzheimer’s disease. Compounds **80a–n** were highly selective towards acetylcholinesterace from electric eel (AChE) and butylcholinesterace from equine serum (BuChE). However, they were more selective towards AChE when compared to BuChE. Compound **80l** was effective at inhibiting AChE with IC_50_ of 12.1 nM while compound **80n** had the highest inhibitory activity against BuChE (IC_50_ = 1.9 nM). The low cinnamic acid inhibitory activity of IC_50_ ˃ 100 nM revealed the requirement of the N-benzyl pyridium moiety for enhanced activity [11].

The methoxy groups present at positions 3 and 4 of the N-benzyl pyridium moiety increased the inhibitory activity of the compound which was significant in compounds **80i–n** when compared to **80b–g**. Activity decreased upon the introduction of fluorine, methyl and bromine on the meta, para position (for example, compound **80a** was more potent than **80g** for AChE). Compounds with different substituents (**80i–n**) had better activity against BuChE which was observed in compound **80h** and compounds with a Br substitution showed higher potency (**80f**, **80g**, **80l** and **80m**) (Table 11). The compounds’ ability to inhibit BuChE was affected more by the substituent size when compared to the electronic properties. Compound **80l** had a dual inhibitory potency and further studies showed that it exhibited good neuroprotective activity against Aβ_1-42_ toxicity in PC12 cells and it can penetrate into the brain, which was observed in PAMPA-BBB assay in vitro making compound **80l** a leading novel compound for the treatment of Alzheimer’s disease [11].

**Scheme 4:**
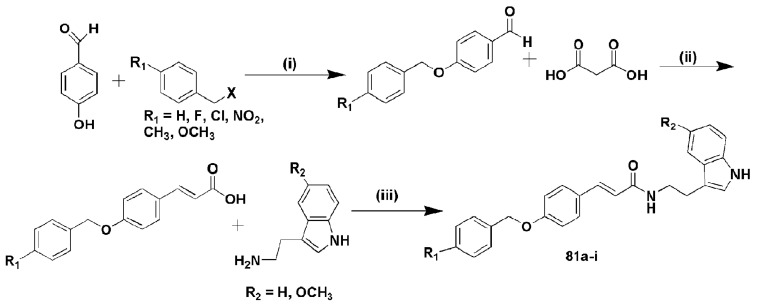
Synthetic route for compounds 81a–l. Reagents and conditions: (i) K_2_CO_3_/DMF/80 °C, (ii) pyrrolidine/pyridine/EtOH/reflux, (iii) EDCl/HOBt/CH_3_CN/rt [56].

Ghafary et al. designed cinnamic acid-tryptamide hybrids (**81a–l**) (Scheme 4) and evaluated their inhibitory activity against acetylcholinesterace (AChE) and butylcholinesterace (BChE) (Table 12) [56]. The presence of either an electron-donating or an electron-withdrawing group on position 4 of the unsubstituted tryptamine derivatives (**81a–f)** reduced the AChE inhibitory activity and the same effect was observed in compounds containing any substituent on the 4-position of the pendant benzyl group in the 5-methoxytryptamine series (**81g–l**), except for **81i**. Replacing fluorine with different electron-withdrawing groups greatly improved BChE inhibition meaning inhibition of BChE potentially depended more on hydrophobic or hydrogen interactions between the chloro or nitro groups with the BChE active site than on the electron-withdrawing effect [56].

For compounds **81g–l**, the presence of an electron-withdrawing group (**81i**; **81j** and **81h**) enhanced the anti-BChE activity with **81g–j** and **81l** being more potent than the standard drug, donepezil. Compound **81d** displayed excellent neuroprotective ability on PC12 neuron cells at 10 µM with cell viability of 62.71. Molecular docking studies showed that **81d** inhibited BChE via a mixed-type mode of inhibition [56].

**Figure 20:**
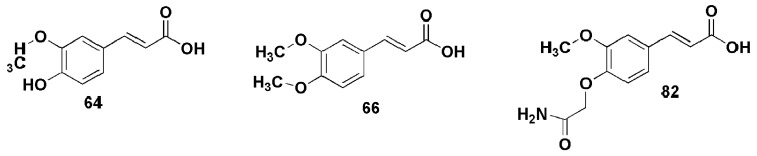
Chemical structures of compounds **64**, **66** and **82** [57,58].

In a recent study, Medvedeva et al. reported the ability of 3-methoxy-4-hydroxycinnamic acid (**64**), 3,4- dimethoxycinnamic acid (**66**) and 3-methoxy-4-acetamidoxycinnamic acid (**82**) (Figure 20) [57] to prevent alpha-synuclein amyloid transformation, thus aiding the treatment of Parkinson’s disease [58]. The IC_50_ values of the compounds were 251 µM for **64**, 50 µM for **82** and 13 µM for **2**. The compounds were also non-toxic towards human neuroblastoma cell line making them very promising for treating synucleinopathies [57].

**Figure 21:**
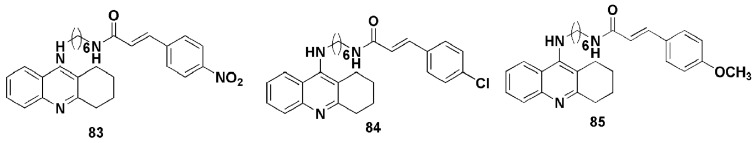
Chemical structures of novel tacrine-cinnamic acid hybrids [59].

Chen et al. evaluated the ability of new tacrine-cinnamic acid hybrids in inhibiting cholinesterase as potential therapeutics for the treatment of Alzheimer’s disease. Only **83**, **84** and **85** (Figure 21) were chosen to validate their inhibitory activities on human cholinesterases (AChE and BuChE). The IC_50_ values of the three compounds on human AChE and BuChE, respectively, were 10.2 nM and 6.3 nM for **83**, 16.5 nM and 5.7 nM for **84** and 15.7 nM and 8.0 nM for **85**. Binding patterns of compounds **83** and **84** with cholinesterases showed π-π stacking interactions, hydrogen bonds and van der Waals forces. At 25 µM, the three compounds inhibited Aβ_1–42_ aggregation with an inhibitory rate between 31.82% and 34.57% when compared to resveratrol (30.36%), a reference compound [59].

Behavioural studies revealed that all the three compounds, **83**–**85** showed enhanced cognitive function in the Institute of Cancer Research (ICR) mice in vivo. Administration of the three compounds caused no remarkable morphological liver changes making them safe and promising compounds for the treatment of Alzheimer’s disease [59].

**Figure 22:**
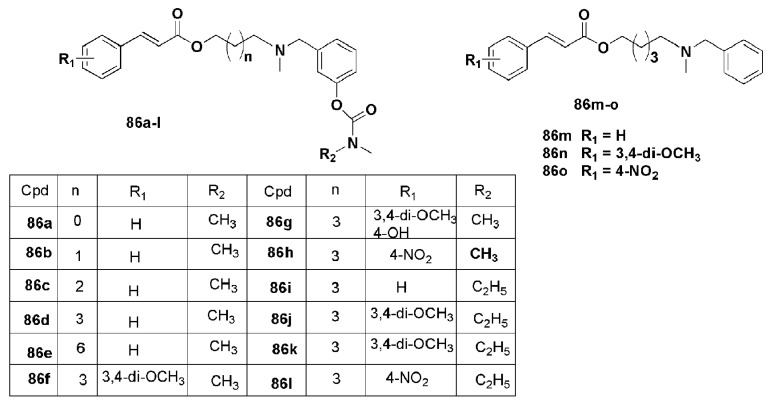
Chemical structures of compounds **86a–o** [60].

Lan et al. reported on ferulic acid derivatives (**86a–o**) (Figure 22) as multi-target inhibitors against Alzheimer’s disease. All the ferulic acid derivatives inhibited eeAChE and eqBuChE with IC_50_ ranging from 0.055 µM to 42.26 µM (Table 13). When evaluated on human ChE (hChE), compounds **86f–h** were potent against both hAChE and hBuChE with inhibitory activity higher than the reference compound, donepezil (Table 13) [60].

The presence of the methoxyl and hydroxyl groups favored the inhibition of Aβ aggregation. Kinetic studies using compound **86g** showed competition for the binding site between the compounds and acetylcholine [60]. The interactions involved π-π stacking, hydrogen bond and cation-π [61]. At concentrations between 6 and 50 µM, compound **86g** exhibited neuroprotective effects. Compounds **86f** and **86g** crossed the BBB thereby reaching the biological targets in the CNS [60].

**Figure 23:**
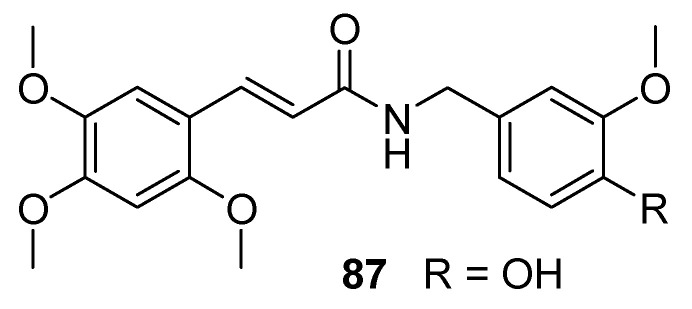
Chemical structure of compound **87** [9].

Zhang et al. synthesized many compounds and found compound **87** (Figure 23) to be the most potent on both the nerve and endothelium cells. Structure–activity relationship analysis showed that the compounds with three methoxyl groups were the most potent indicating the role played by the methoxyl group in optimizing the compound’s activity [9].

Compound **87** exhibited high neuroprotective ability against H_2_O_2_-induced damage with EC_50_ values of 3.26 µM and 2.41 µM in HBMEC-2 and SH-SY5Y cells, respectively. Compound **87** showed potential in inhibiting the apoptosis of HBMEC-2 and SH-SY5Y cells induced by H_2_O_2_, and also inhibited neuronal apoptosis and promoted angiogenesis [9].

**Figure 24:**
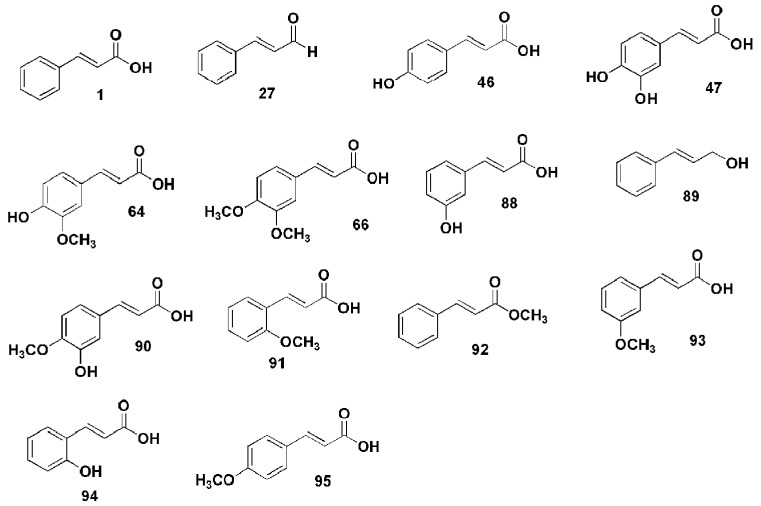
Structures of cinnamic acid derivatives effective against Protein Tyrosine Phosphatase 1B (PTP1B) [13].

Adisakwattana et al. evaluated the in vitro inhibitory effects of cinnamic acid derivatives (**1**, **27**, **46**, **47**, **64**, **66**, **88**–**95**) (Figure 24) against Protein Tyrosine Phosphatase 1B (PTP1B). Results showed that replacing the carboxyl group of cinnamic acid (**1**) (16.0% inhibition) with alcohol, aldehyde and methyl ester groups (**89, 27** and **92**) (12.5–18.0% inhibition) did not increase the PTP1B inhibition percentage. Compounds **46** and **94** exhibited the highest inhibition of 39.4% and 35.9%, respectively, showing the role played by the hydroxyl group on the ortho- or para-positions. Introducing two moieties on cinnamic acid resulted in compounds with better inhibition (21.5–24.4%) when compared to cinnamic acid except for compound **64** [13].

The most potent compounds with effective neurological activity are compounds **86f–h**. They were active against both hAChE and hBuChE when compared to donepezil in vitro [60]. There is a pressing need for these compounds to be evaluated in vivo.

## 6. Cinnamic Derivatives with Antidiabetic Activity

Diabetes mellitus is characterized as a metabolic disorder that results from a defect in insulin action, secretion or both [62]. The International Diabetes Federation estimated the global prevalence of diabetes mellitus to increase to 591 million by 2035 from 382 million reported in 2013. The inability to regulate blood glucose also causes many complications such as angiopathy, retinopathy and neuropathy [63]. The inhibitory effect of cinnamic acid derivatives on Protein Tyrosine Phosphatase 1B (PTP1B) has been one of the several ways to regulate blood glucose [13].

Amalan et al. also reported the ability of compound **46** in lowering blood glucose and improving insulin levels [64]. Compounds **94** and **89** (Figure 24) inhibited p-NPP hydrolysis catalysed by PTP1B with IC_50_ values of 137.74 and 168.40 nM. Kinetic studies proved compounds **89** and **94** to be non-competitive inhibitors with the ability of being developed into highly selective PTP1B inhibitors [64], thus cinnamic acid derivatives can be further explored as PTP1B inhibitors for the treatment of diabetes [13]. Hypochlorous acid (HOCl) and H_2_O_2_ scavenging assay results showed compound **95** has the antioxidant capability to capture free radicals and reactive species such as O_2_**^·-^** and HOCl/OCl^-^ [64] which causes damage to the organs in the body [65].

Adisakwattana et al. evaluated compounds **1**, **46**–**47**, **64**, **66**, **88**–**91** and **93**–**95** (Figure 24) for their inhibitory activity against intestinal α-glucosidase (maltase and sucrose). The most effective compounds (**64**, **90** and **47**) exhibited a non-competitive inhibition, better on sucrase when compared to maltase (Table 14), although these compounds were less potent than acarbose with IC_50_ values of 2.2 and 4.8 µM for maltase and sucrase, respectively. Combining compounds **64**, **90** and **47** with acarbose produced additive inhibition resulting in reduced postprandial hyperglycemia and hyperinsulinemia in type 2 diabetes [66].

**Figure 25:**
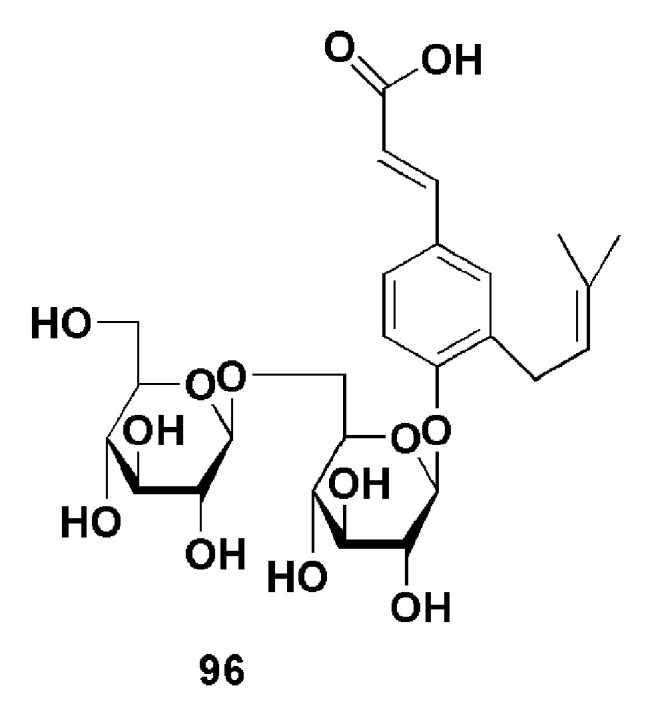
Chemical structure of dissectumoside, **96** [67].

Wang et al. isolated a cinnamic acid glycoside derivative, dissectumoside (**96**), from the roots of *H. dissectum* (Figure 25). When evaluated for its in vitro triglycerides accumulation activity in 3T3-L1 adipocytes, compound **96** increased activity from 7.8% at 1 µM through 32.1% at 10 µM to 40.1% at 30 µM. The authors reported that compound **96** provided antidiabetic effect [67] as previously reported [68] due to its ability to effectively transport glucose from the medium into adipocytes and triglycerides [67].

**Figure 26:**
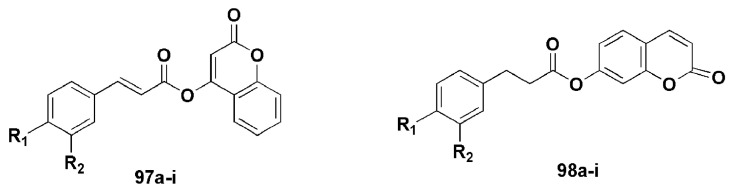
Chemical structures of coumarin derivatives (**97a–i** and **98a–i**) [69].

Xu et al. evaluated the activity of coumarin derivatives (**97a–i** and **98a–i**) (Figure 26) as α-glucosidase inhibitors with both the 97 series and the 98 series derivatives having the same substituents. Compounds **98a–i** were less potent with **98b** having the highest inhibition of 54.745 at 100 µM and IC_50_ of 94.52 µM, the rest had inhibition less than 50% and IC_50_ ˃100 µM [69].

Since compounds **97a–i** were better inhibitors (Table 15) than **98a–i**, it showed that conjugating cinnamic acid with 4-hydroxycoumarin was better than with 7-hydroxycoumarin. Compound **97b** exhibited the most effective inhibition activity of 89.92% and IC_50_ of 12.98 µM suggesting that the presence of a methyl group, which is an electron-donating group, favored the withdrawing group (**97e–i**). The two most potent compounds, **97a** and **97b**, showed that their α-glucosidase inhibition was reversible. Compound **97b** fitted well into the active site of α-glucosidase forming a hydrogen bond with the LYS293 amino acid sequences [69].

## 7. Cinnamic Acid Derivatives with Antioxidant and Anti-Inflammatory Activity and in Cosmetics

Biomolecular oxidations of reactive oxygen and nitrogen species create oxidative stress. Free radicals produce damaged membranes, produce DNA nicks and inflict other oxidative injuries. Accumulation of free radicals activates proto oncogenes causing carcinogenesis [70]. There is a need to scavenge free radicals in the body to minimize the damage inflicted by free radicals. Cinnamic acid derivatives are known to have antioxidant properties [4]. Three mechanisms are reported in phenolic compounds during free radical scavenging. The first one involves a hydrogen atom being abstracted from the antioxidant by the radical. The second step involves the formation of a radical cation ArOH^+•^ through the transfer of an electron to the free radical from the antioxidant and the deprotonation yielding ArO^•^ radical and ROH. In the last step, the phenolic compound is deprotonated and there is an electron transfer from the phenoxide anion to RO^•^ to yield phenoxyl radicals [71].

**Figure 27:**
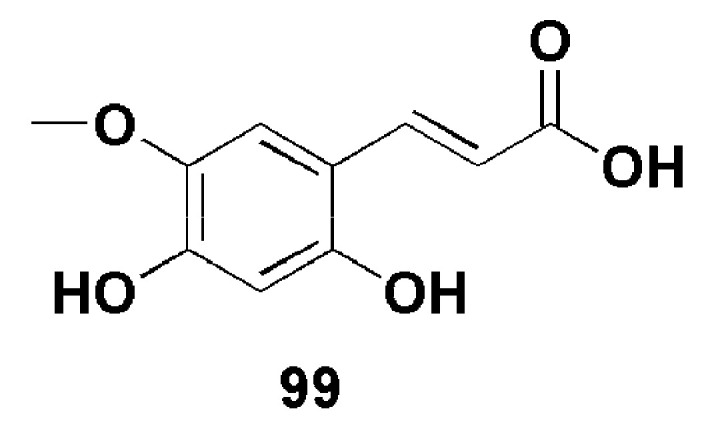
Structure of compound **99** [72].

Muhammad et al. isolated a new bioactive compound (**99**) (Figure 27) from the plant, *Viola betonicifolia*. At the concentration range of 2–50 µg/mL, compound **99** inhibited DPPH (diphenyl-2-picryl hydrazyl) free radicals with IC_50_ of 124 µM which was higher than for common antioxidants α-tocopherol (IC_50_ of 96 µM) and vitamic C (IC_50_ of 90 µM). As an anti-glycating agent, compound **99** showed moderate activity with IC_50_ of 355 µM when compared to the IC_50_ of rutin, a standard drug which was 294 µM. Thus, due to its free radical scavenging ability, compound **99** has the potential to delay and prevent diabetic complications [72].

**Figure 28:**
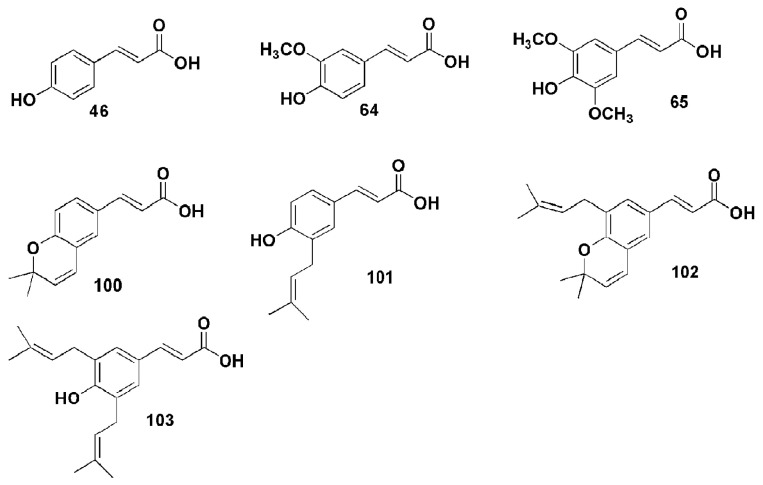
Chemical structures of compounds **46**, **64**, **65**, **100**–**103** [73].

Kortenska et al. reported the antioxidant activity of compounds **46**, **64**, **65**, **100**–**103** (Figure 28) in pure triacylglycerols of sunflower oil (TGSO) at 80 °C. Compound **101** showed the highest antioxidant activity at 0.1 mM [73]. The reaction of a phenol antioxidant with lipid radicals produces phenoxyl radicals which are stabilized by unpaired electrons delocalized around the aromatic ring [61]. Replacing hydrogen atoms in the o-position with prenyl groups increased the electron density of the OH-moiety through inductive effects thereby enhancing its reactivity towards lipid radicals [74]. This resulted in compounds **101** and **102** being more active than compound **46** [73].

Transformation of the phenol in compounds, **100** and **102** led to the loss of antioxidant activity as there was no hydrogen donating group left. The antioxidant efficiency was found to decrease in the order: **65** ˃ **101** ˃ **103** ˃ **64** ˃ **46** ≈ **100** ≈ **102**. Inhibition degree (measure of antioxidant strength), that is the number of times the antioxidant shortened the oxidation chain length, followed the sequence α-tocopherol ˃ **101** ˃ **103** ˃ **65** ˃ **64** ˃ **46** ≈ **100** ≈ **102**. The antioxidant activity, calculated as the ability of an inhibitor to terminate the oxidation chain and also the decreased oxidation rate during induction period, followed the sequence: α-tocopherol ˃ **101** ˃ **103** ˃ **65** ˃ **64** ˃ **46** ≈ **100** ≈ **102 [73].**

**Scheme 5:**
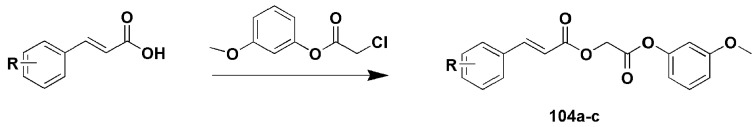
Synthesis of compounds (**104a–c**) [75].

Nazir et al. synthesized cinnamic acid derivatives (**104a–c**) (Scheme 5) with tyrosinase inhibition activity for hyperpigmentation treatment. Compound **104c** (IC_50_ = 5.7 µM) was the most potent compound (Table 16) and kinetic studies revealed that compound **104c** inhibited tyrosinase [75].

Treatment of B16F10 melanoma cells with compound **104c** (30 µg/mL) showed cell viability of higher than 99%. Compound **104c** proved a better inhibitor than kojic acid since 125 µg/mL of kojic acid was needed to achieve the same inhibition effected by 30 µg/mL of compound **104c**. Thus compound **104c** is a promising melanogenic regulator [75].

**Figure 29:**
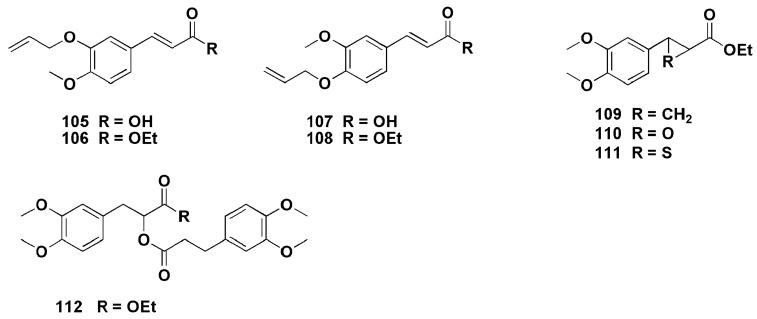
Chemical structures of compounds **105**–**112** [12].

Gießel et al. synthesized cinnamic acid derivatives (**105**–**112**) (Figure 29) for use in flavors and fragrances. When evaluated for their biological activity, the compounds were weak inhibitors of both eeAChE and eqBuAChE with the highest inhibition being 45.5%. Cytotoxic studies against several human tumour cell lines and non-malignant mouse fibroblasts showed the compounds to be non-cytotoxic since they all had EC_50_ values of more than 30 µM, thus can be safely used as cosmetics and flavoring. The compounds had a typical vanillin smell suggesting their potential use in fragrances and flavor [12].

Taofiq et al. evaluated the anti-inflammatory and anti-tyrosinase activities of p-coumaric acid (compound **46**). Its anti-inflammatory activity was measured to be EC_50_ of 152 µg/mL and anti-tyrosinase activity of EC_50_ ˃ 2.0 mg/mL. [76]. The ability of compound **46** to inhibit tyrosinase was credited to its ability to reduce microphthalmia-associated transcription factor (MITF) [77] and tyrosinase mRNA expression as high as 73% and 82%, respectively. The results showed that compound **46** has the potential to be used together with other bioactive ingredients for protection against hyperpigmentation [76]. Gunia-Krzyzak et al. reported compound **46**-containing cream that decreased erythema and pigmentation when applied daily on the foreskin arm [78].

Lee et al. reported the ability of hydroxycinnamic acids (cinnamic acid (**1**), p-coumaric acid (**46**), caffeic acid (**47**) and chlorogenic acid (**33**) on reducing furan and trapping α-dicarbonyl compounds, which are possible human carcinogens. [79]. Addition of water-soluble antioxidants such as caffeic acid, chlorogenic acid and ferulic acid has been found to reduce levels of furan derivatives [80]. When compound **47** was added to canned-coffee model systems (CCMS) containing glyoxal (GO), furan was reduced from 59.76 µg/L to 48.31 µg/L while compound **51** reduced it to 41.38 µg/L.

Compound **47** reduced furan in methyglyoxal (MGO) from 45.79 µg/L to 35.41 µg/L while compound **33** reduced it to 32.65 µg/L. The results showed the capability of the compounds to suppress furan formation in CCMS. Compounds **47** and **33** showed better GO and MGO trapping efficiency when compared to compounds **1** and **46**. This was attributed to the presence of two hydroxyl groups on the aromatic ring trapping the dicarbonyls thereby causing the reduction of furan concentration in CCMS [79].

**Figure 30:**
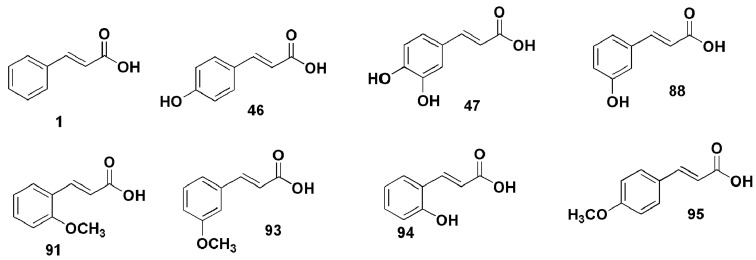
Chemical structures of compounds **1**, **46**, **47**, **88**, **91** and **93**–**95** [81].

Garcia-Jimenez et al. reported the catalysis and inhibition of tyrosinase when subjected to different cinnamic acid derivatives (**1**, **46**, **47**, **88**, **91** and **93**–**95**) (Figure 30). Results of the compounds in the presence of 4-tert-butylcatechol (TBC) showed that compounds **1**, **91**, **93**, **94** and **95** are competitive inhibitors whereas compounds **46**, **47** and **88** are substrates of tyrosinase. The inhibition constants were affected by the ability of the substituent group to increase the charge on the benzene ring [81].

The methoxy group, OCH_3_ is an electron donor and it increased the charge on the benzene ring making the inhibition constant to be in the order **95** ˃ **93** ˃ **91** ˃ **1**. Tyrosinase could not hydroxylate compound **94** but was able to hydroxylate compound **88** because compound **94** has a great distance for ligand hydroxylation while compound **88** has a distance feasible for hydroxylation [81].

**Scheme 6:**
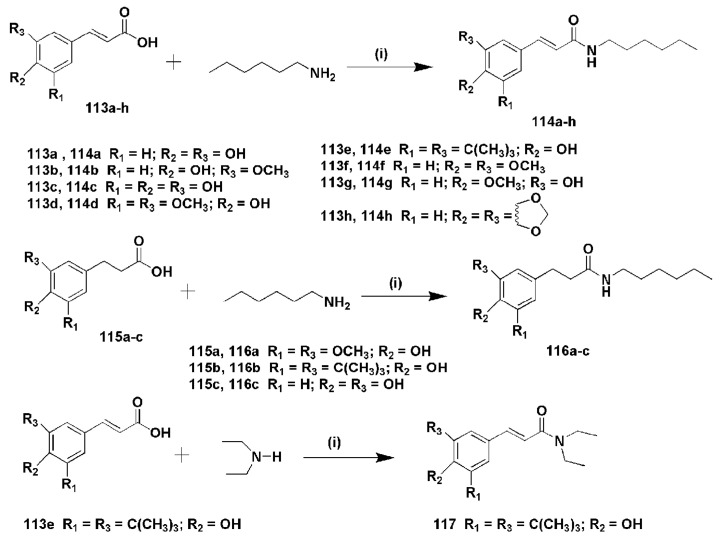
Synthesis of compounds **113a–h**, **114a–h**, **115a–c**, **116a–c** and **117**. Reagents and conditions: (i) Dimethylformamide (DMF), Triethylamine (TEA), Benzotriazol-1-yloxy)tris (dimethylamino) phosphonium hexafluorophosphate(BOP), Dichloromethane (DCM), room temperature (rt) [82].

Ribeiro et al. evaluated the inhibition of cyclooxygenase enzymes (COX-1 and COX-2) by synthesized cinnamic acid derivatives (**113a–h**, **114a–h**, **115a–c**, **116a–c** and **117**) (Scheme 6). Table 17 showed the most important structure–activity relationship for COX-1 inhibition with the presence of the di-substituted aromatic rings having one hydroxyl group and one methoxyl group or two hydroxyl groups, an amide group and the absence of an α,β-double bond [82].

These aforementioned features gave compounds **114e** (IC_50_ = 24 µM and 65% inhibition), **114g** (IC_50_ = 9 µM and 85% inhibition) and **116c** (IC_50_ = 15 µM and 84% inhibition). Compounds **116a** and **117** were also good COX-1 inhibitors meaning a tri-substituted aromatic ring with one hydroxyl group and two methoxyl groups and no α,β-double bond made compound **116a** effective. The best COX-1 inhibitor was compound **117** (IC_50_ = 4.3 µM) meaning the 3,5-di-*tert*-butyl-4-hydroxyl aromatic substitution, the presence of tertiary diethylamide group and an α,β-double bond contributed to its biological activity [82].

Compounds **113e**, **114a**, **114e**, **116c**, and **117** appeared to be the best COX-2 inhibitors with IC_50_ values of 3.0, 12.7, 2.4, 13.4, and 1.09 µM, respectively. The structure–activity relationship of the compounds revealed the presence 3,5-di-*tert*-butyl-4-hydroxyl aromatic substitution, tertiary diethylamide group and an α,β-double bond (compound **132**) and a disubstituted aromatic ring with two hydroxyl groups, an amide group and an α,β-double bond influenced the biological activity of these compounds. Since compounds **113e**, **114e**, **116d** and **117** were the most potent compounds against COX-2, they are suggested as potential non-steroidal anti-inflammatory drugs [82].

**Figure 31:**
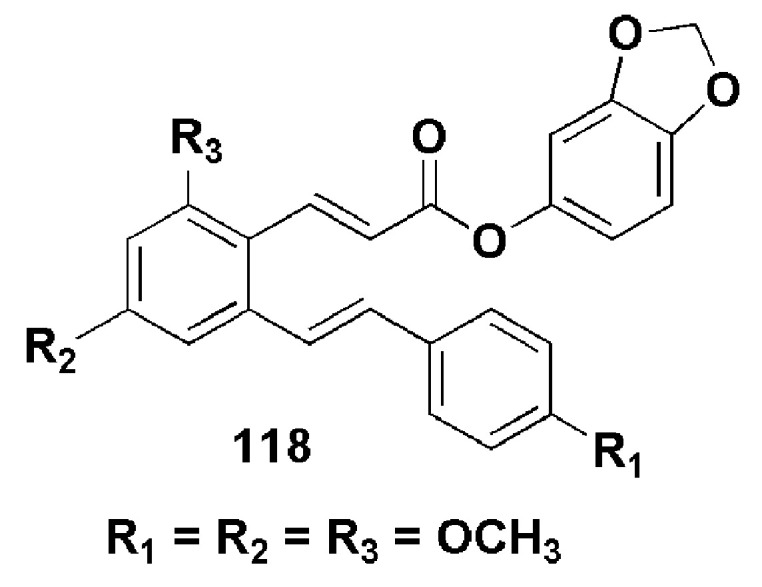
Chemical structure of compound **118** [83].

Ruan et al. reported the potency of a resveratrol-based cinnamic acid derivative, **118** (Figure 31). Compound **118** inhibited LPS-mediated expression of inducible nitric oxide synthase (iNOS) and COX-2 in RAW264.7 cells when analysed by Western blot [83].

Inflammation-related diseases are characterized by the expressions of iNOS and COX-2. Compound **188** suppressed the production of NO in LPS-induced RAW264.7 cells in vitro. Furthermore, it inhibited LPS-induced protein expression and immunofluorescence study showed that the compound slightly lowered the activation p65 in the nuclei. The anti-inflammatory role of the compound is partially attributed to its inhibitory effect on the NF-κB signaling pathway LPS-induced RAW 264.7 cells. [83].

## 8. Conclusions

Research reports have shown that derivatives of cinnamic acid are active in vitro when compared to some of the currently used drugs such as the anticancer, antimicrobial drugs etc. which were used as control. The antimicrobial, anticancer and anti-oxidant activity of the derivatives was influenced by the nature and position of the substituent groups on the phenyl ring. The length of the linker also influenced the biological activity of the derivatives.

Furthermore, the toxicological studies performed on some of the derivatives revealed the non-toxic effects of the compounds suggesting their suitability for applications in vivo. However, the major draw-back pertaining to the synthesized derivatives is that most of them lack in vivo data. Thus, adding to the in vitro analyses that have been carried on most of the potent derivatives, there is a need to proceed to in vivo studies. More research on the development of cinnamic derivatives has the potential to result in potent compounds that will successfully reach clinical trials.

## Figures and Tables

**Table 1:** Comparing percentage fungal growth of the three fungi when treated with 0.5 mmol/L of cinnamic acid derivatives (**3**–**9**) [22].

Compounds	%Fungal Growth in 0.5 mmol/L of Inhibitor		
	*C. lunatus*	*A. niger*	*P. ostreatus*
**3**	22	85	38
**4**	25	66	43
**5**	30	28	29
**6**	34	109	00
**7**	42	84	62
**8**	58	60	67
**9**	63	50	53
**control**	90	109	55

**Table 2:** In vitro anti-tubercular activity of compounds **10**–**26** against *M.tb* H37Ra on day 8 [24].

Compound	IC_50_ (µg/mL)	Cpd.	IC_50_ (µg/mL)
**1**	0.06	**18**	˃30
**10**	1.42	**19**	11.01
**11**	˃30	**20**	0.045
**12**	˃30	**21**	0.56
**13**	˃30	**22**	˃30
**14**	4.53	**23**	0.56
**15**	9.91	**24**	˃30
**16**	2.35	**25**	˃30
**17**	0.36	**26**	1.56

**Table 3:** Minimum inhibitory concentration (MIC) of representative biocides and derivatives against *E. coli*, *S. aureus* and *E. hirae* [16].

Compounds	*E. coli* (mM)	*S. aureus* (mM)	*E. hirae* (mM)
Sodium hypochlorite	5	8	5
Triclosan	0.003	0.003	0.02
**27**	3	3–5	5–8
**28**	20–25	˃25	˃25
**29**	15–20	15–20	20–25
**30**	15–20	15–25	18–25
**31**	˃13	˃13	˃13
**32**	˃25	˃25	˃25

**Table 4:** IC_50_ values of oleanolic acid (OA), glycyrrhetinic acid (GA) and their derivatives towards L-O2, MCF-7 and HeLa cells [5].

Compounds	IC_50_ (µM)			Compounds	IC_50_ (µM)		
	L-O2	MCF-7	HeLa		L-O2	MCF-7	HeLa
**OA**	32.9	˃100	˃100	**44m**	˃100	38.89	56.29
**GA**	˃100	64.16	98.99	**44o**	˃100	˃100	1.35
**43d**	˃100	32.49	1.55	**45c**	10.19	˃100	˃100
**43a**	˃100	˃100	˃100	**45e**	˃100	36.84	˃100
**43e**	˃100	1.79	˃100	**45f**	˃100	˃100	˃100
**44h**	˃100	˃100	˃100	**45i**	˃100	46.53	˃100

**Table 5:** In vitro antiproliferative activity of compounds **53a–o** as IC_50_ (µM) [31].

Cpd.	MCF-7	A549	H157	HepG2	HL-60	Cpd.	MCF-7	A549	H157	HepG2	HL-60
**53a**	˃50	˃50	˃50	˃50	˃50	**53i**	˃50	˃50	˃50	˃50	˃50
**53b**	˃50	˃50	3.72	5.46	˃50	**53j**	˃50	˃50	˃50	˃50	10.19
**53c**	4.16	˃50	24.50	6.55	˃50	**53k**	˃50	˃50	˃50	˃50	˃50
**53d**	8.85	30.98	21.33	22.59	15.68	**53l**	˃50	˃50	˃50	5.5	˃50
**53e**	˃50	˃50	24.54	3.81	˃50	**53m**	6.55	36.20	30.67	˃50	11.26
**53f**	4.91	3.22	4.28	3.45	6.87	**53n**	˃50	˃50	˃50	˃50	˃50
**53g**	6.46	5.16	3.77	8.86	3.51	**53o**	˃50	˃50	˃50	3.10	˃50
**53h**	6.11	˃50	17.98	5.22	25.64	**Dox**	1.51	0.31	0.48	1.82	0.91

**Table 6:** IC_50_ values of compounds **55g,h**, **55k,l**, and **55o,p** against drug-sensitive HCC Bel7402 and drug-resistant HCC Bel7402/5-FU cells [30].

Compounds	IC_50_(µM)	
	Bel7402	Bel7402/5-FU
SAHA	4.72	9.83
5-FU	15.6	61.7
**55g**	1.03	2.17
**55h**	2.15	2.90
**55k**	3.24	4.38
**55l**	2.71	3.06
**55o**	1.97	3.66
**55p**	0.85	2.09

**Table 7:** Antiproliferative activities and anti-tubulin activities of **56a**–**58e** against MCF-7 cells [47].

Cpd.	n	R	MCF-7 IC_50_ (µg/mL)	ITP IC_50_ (µM)	Cpd.	n	R	MCF-7 IC_50_ (µg/mL)	ITP IC_50_ (µM)
**56a**	1	H	0.17	0.88	**57d**	2	Cl	51.31	87.92
**56b**	1	CH_3_	48.95	40.85	**57e**	2	Br	˃100	˃100
**56c**	1	F	74.05	76.90	**58a**	3	H	53.87	49.01
**56d**	1	Cl	47.27	94.72	**58b**	3	CH_3_	˃100	˃100
**56e**	1	Br	89.01	51.59	**58c**	3	F	˃100	˃100
**57a**	2	H	2.16	5.47	**58d**	3	Cl	˃100	˃100
**57b**	2	CH_3_	˃100	93.33	**58e**	3	Br	˃100	˃100
**57c**	2	F	52.13	27.46	**Colchicine**			0.33	1.36

**Table 8:** IC_50_ (µM) values for compounds **35**–**37** after in vitro screening against erythrocytic stage of *P. falciparum* (Pf3D7 and PfDd2 strains) and hepatic stages of *P. berghei* [33].

Cpd	Pf3D7	PfDd2	*P. berghei*	Cpd	Pf3D7	PfDd2	*P. berghei*
**35a**	1.26	23.76	2.62	**36g**	0.15	0.69	-
**35b**	0.61	16.61	-	**36h**	0.13	0.46	-
**35c**	0.50	1.57	-	**36i**	0.33	3.06	0.66
**35d**	0.89	2.70	-	**37a**	0.56	1.59	-
**35e**	0.87	2.21	-	**37b**	0.13	0.16	0.4
**35f**	0.65	2.55	-	**37c**	0.44	0.65	-
**35g**	0.44	1.87	1.12	**37d**	2.50	3.23	-
**35h**	0.56	2.26	1.10	**37e**	0.62	8.00	-
**35i**	0.77	2.82	-	**37f**	1.07	17.94	-
**36a**	0.26	1.09	-	**37g**	0.25	3.28	-
**36b**	0.43	0.69	-	**37i**	1.34	4.02	-
**36c**	0.16	0.47	-	**CQ**	0.003	0.20	-
**36d**	0.20	1.19	-	**Harmine**	8.25	˃27.7	-
**36e**	0.20	0.84	-	**Primaquinee**	-	-	8.4
**36f**	0.34	1.01	-				

**Table 9:** % toxicity of the compounds on *Leishmania* intracellular amastigotes after 48 h. All compounds were 10 µM [3].

Compounds	% Toxicity	Cpd.	% Toxicity
**61a**	43.87	**62c**	59.10
**61b**	80.47	**62d**	80.12
**61c**	59.53	**63a**	45.10
**61d**	50.14	**63b**	67.84
**62a**	19.91	**63c**	69.24
**62b**	86.32	**63d**	19.67

**Table 10:** Mean mortality percentages of *Psoroptes cuniculi* when incubated with 0.5 mg/mL and 0.25 mg/mL of different compounds [52].

Cpd.	Mean Mortality %		LC_50_ [mM] (24 h)	LT_50_ (h)
	0.5 mg/mL	0.25 mg/mL		
**67**	100.0	78.3	1.07	13.3
**68**	100.0	98.3	0.56	12.0
**69**	90.0	76.0	0.87	13.9
**70**	84.0	68.0	0.74	15.2
**71**	100.0	100.0	0.45	9.8
**72**	100.0	80.0	0.82	15.6
**73**	100.0	84.0	0.64	6.8
**74**	100.0	100.0	0.36	2.6
**75**	98.0	66.0		
**76**	65.0	16.7		
**77**	38.3	-		
**78**	26.0	-		
**79**	94.0	44.0		
**Ivermectin**	75.0	68.3	0.28	8.9

**Table 11:** Inhibition concentration (IC_50_) of ChEs activity in nM [11].

Compound	eeAChE	eqBuChE	Cpd.	eeAChE	eqBuChE
**80a**	54.1	6.3	**80i**	51.4	2.7
**80b**	102.6	8.1	**80j**	90.7	3.8
**80c**	1263.8	25.0	**80k**	29.6	3.1
**80d**	93.5	7.4	**80l**	12.1	2.6
**80e**	90.8	10.0	**80m**	92.6	2.5
**80f**	153.6	2.5	**80n**	50.3	1.9
**80g**	1450.5	2.1	**Cinnamic acid**	˃100,000	˃100
**80h**	135.2	4.5	**Donepezil**	40.2	4.5

**Table 12:** Cholinesterace inhibitory activity IC_50_ (µM) of compounds **81a–l** [56].

Cpd	R_1_	R_2_	AChE	BChE	Cpd	R_1_	R_2_	AChE	BChE
**81a**	H	H	14.93	2.12	**81h**	F	OCH_3_	29.67	5.89
**81b**	F	H	32.17	9.36	**81i**	Cl	OCH_3_	13.42	2.86
**81c**	Cl	H	24.7	0.89	**81j**	NO_2_	OCH_3_	31.45	4.28
**81d**	NO_2_	H	34.49	0.55	**81k**	CH_3_	OCH_3_	31.85	12.25
**81e**	CH_3_	H	39.1	7.73	**81l**	OCH_3_	OCH_3_	25.11	7.69
**81f**	OCH_3_	H	34.95	2.51	**Donepezil**			0.027	7.79
**81g**	H	OCH_3_	14.18	6.63					

**Table 13:** IC_50_ values of compounds **86f–h** on human AChE (hAChE) and BuChE (hBuChE) [60].

Cpd.	hAChE (nM)	HBuChE (µM)
**86f**	16.5	0.76
**86g**	19.7	0.59
**86h**	13.7	0.66
Donepezil	30.2	8.7

**Table 14:** IC_50_ values for maltase and sucrase inhibition by compounds **47**, **64** and **90** [67].

Compounds	Maltase	Sucrase
**47**	0.74	0.49
**64**	0.79	0.45
**90**	0.76	0.45

**Table 15:** Inhibitory Activity of Compounds **97a–i** on α-glucosidase [69].

Compounds	97a	97b	97c	97d	97e	97f	97g	97h	97i
R_1_	H	CH_3_	OCH_3_	F	Cl	Br	CF_3_	OH	OH
R_2_	H	H	H	H	H	H	H	H	OCH_3_
% inhibition	83.51	89.92	73.15	68.50	33.10	60.99	41.87	33.19	19.51
IC_50_ (µM)	19.64	12.98	36.88	41.73	˃100	69.30	˃100	˃100	˃100

**Table 16:** Substituents, mushroom tyrosinase inhibitory activity, IC_50_ (µM) and kinetics of compounds **104a–c** [75].

Cpd	R	IC_50_ (µM)	K_f_ (µM)
**104a**	H	39.4	-
**104b**	2-OH	172.8	-
**104c**	4-OH	5.7	11
**Kojic acid**		16.7	n.d.

n.d., not determined. K_f_ is the kinetic reaction constant.

**Table 17:** IC_50_ and inhibition % of PGE_2_ production via COX-1 and COX-2, in human whole blood [82].

Cpd.	COX-1		COX-2		Cpd.	COX-1		COX-2	
	IC_50_ (µM)	Inhibition (%)	IC_50_ (µM)	Inhibition (%)		IC_50_ (µM)	Inhibition (%)	IC_50_ (µM)	Inhibition (%)
**113a**	N.A.	N.A.	N.A.	N.A.	**114g**	9	85	21	79
**113b**	N.A.	N.A.	N.A.	N.A.	**114h**	N.A.	N.A.	N.A.	N.A.
**113c**	˃100	41	N.A.	N.A.	**115b**	97	57	N.A.	N.A.
**113e**	˃100	39	3.0	93	**116a**	11	82	62	90
**114a**	24	82	12.7	99.0	**116b**	˃100	24	76	57
**114b**	14	81	20	98.3	**116c**	15	84	13.4	98.0
**114c**	87	60	30	74	**117**	4.3	79	1.09	85
**114d**	45	82	19	91	**Ind.**	-	91	-	-
**114e**	24	65	2.4	99.4	**Cel.**	-	-	-	71
**114f**	N.A.	N.A.	N.A.	N.A.					

N.A.—Non active up to highest tested concentration of 100 µM. All the compounds were tested at 100 µM except for compound **117** tested at 12.5 µM for COX-1 and 3.1 µM for COX-2. The two reference drugs were tested at 1 µM. Where Ind. and Cel. represent Indomethacin and Celecoxib, respectively.

## Abbreviations

Cpd.	Compound
IC_50_Half	maximal inhibitory concentration
EC_50_	Half maximal effective concentration
HSV	Herpes simplex virus
HIV	Human immunodeficiency virus
DMSO	Dimethyl sulfoxide
TEA	Triethylamine
DCM	Dichloromethane
EDCl	1-Ethyl-3-(3-dimethylaminopropyl)carbodiimide
DMAP	4-Dimethylaminopyridine
DMF	D imethylformamide
HOBt	Hydroxybenzotriazole
CNS	Central Nervous System
BBB	Blood-Brain Barrier
AKT	Protein kinase B
ERK	extracellular signal-regulated kinase
